# Exosomal microRNAs derived from colorectal cancer-associated fibroblasts: role in driving cancer progression

**DOI:** 10.18632/aging.101355

**Published:** 2017-12-28

**Authors:** Rahul Bhome, Rebecca W. Goh, Marc D. Bullock, Nir Pillar, Stephen M. Thirdborough, Massimiliano Mellone, Reza Mirnezami, Dieter Galea, Kirill Veselkov, Quan Gu, Timothy J. Underwood, John N. Primrose, Olivier De Wever, Noam Shomron, A. Emre Sayan, Alex H. Mirnezami

**Affiliations:** ^1^ Cancer Sciences, University of Southampton, Somers Building, Southampton General Hospital, Southampton SO16 6YD, UK; ^2^ University Surgical Unit, University of Southampton, Southampton General Hospital, Southampton SO16 6YD, UK; ^3^ Department of Cell and Developmental Biology, Sackler Faculty of Medicine, Tel Aviv University, Tel Aviv 69978, Israel; ^4^ Department of Surgery and Cancer, Imperial College London, Sir Alexander Fleming Building, London SW7 2BB, UK; ^5^ University of Glasgow Centre for Virus Research, 117 Sir Michael Stoker Building, Glasgow G61 1QH, UK; ^6^ Department of Experimental Cancer Research, Ghent University, Radiotherapiepark, 9000 Ghent, Belgium

**Keywords:** cancer-associated fibroblasts, exosomes, microRNA, stroma, colorectal cancer

## Abstract

Colorectal cancer is a global disease with increasing incidence. Mortality is largely attributed to metastatic spread and therefore, a mechanistic dissection of the signals which influence tumor progression is needed. Cancer stroma plays a critical role in tumor proliferation, invasion and chemoresistance. Here, we sought to identify and characterize exosomal microRNAs as mediators of stromal-tumor signaling. *In vitro*, we demonstrated that fibroblast exosomes are transferred to colorectal cancer cells, with a resultant increase in cellular microRNA levels, impacting proliferation and chemoresistance. To probe this further, exosomal microRNAs were profiled from paired patient-derived normal and cancer-associated fibroblasts, from an ongoing prospective biomarker study. An exosomal cancer-associated fibroblast signature consisting of microRNAs 329, 181a, 199b, 382, 215 and 21 was identified. Of these, miR-21 had highest abundance and was enriched in exosomes. Orthotopic xenografts established with miR-21-overexpressing fibroblasts and CRC cells led to increased liver metastases compared to those established with control fibroblasts. Our data provide a novel stromal exosome signature in colorectal cancer, which has potential for biomarker validation. Furthermore, we confirmed the importance of stromal miR-21 in colorectal cancer progression using an orthotopic model, and propose that exosomes are a vehicle for miR-21 transfer between stromal fibroblasts and cancer cells.

## INTRODUCTION

Colorectal cancer (CRC) poses a substantial public health problem, with global incidence set to eclipse two million by 2030 [1]. In Europe, CRC represents the second highest cause of cancer-related death, healthcare expenditure, and loss of productivity [2]. The principal cause of mortality from CRC is metastasis. Despite advances in surgical and chemotherapeutic treatment options for metastatic CRC, the majority of patients remain incurable, with a median survival of less than two years [3]. This highlights the need to identify novel therapeutic targets and better markers of metastatic capability, enabling stratification of high-risk patients for treatment intensification and less radical treatment for lower risk disease.

The consensus view of a tumor resembling an organ has highlighted the critical role of the tumor microenvironment in recent years [4]. The shift in focus has revealed that stromal cells such as cancer-associated fibroblasts (CAFs) are key players in modulating tumor progression [5-7]. Moreover, a dynamic and reciprocal interaction between cancer and stromal cells has been demonstrated, highlighting the profound impact that stromal cells have on proliferation, angiogenesis, invasion, metastasis and chemoresistance, thereby promoting cancer progression through multiple pleiotropic mechanisms [5-7]. It is therefore understandable, that a significant number of genes which stratify better and worse prognoses, are defined by the stromal compartment [8].

MicroRNAs (miRNAs) are small non-coding RNAs that negatively regulate gene expression and have been shown to control many cellular genes and pathways. Recent work by our group has revealed that deregulated miRNA expression in CRC stroma is of clinical significance [9, 10]. One miRNA taken forward was miR-21, an oncogenic miRNA overexpressed in several solid tumors, which regulates the tumor suppressor PDCD4 in CRC [11, 12]. Whilst previous studies identified miR-21 upregulation in CRC, these considered whole-tissue only [13, 14]. In contrast, we and others have shown that miR-21 is overexpressed in CRC stroma by CAFs, stratifying patients with early-stage CRC for recurrence, disease free survival and overall survival [10]. Mechanistically, we demonstrated that over-expression of miR-21 in CAFs promotes increased invasiveness, proliferation and chemotherapy resistance in surrounding tumor cells by paracrine signaling [9]. Clearly then, it is important to elucidate mechanisms by which stromal gene expression is relayed to cancer cells. Exosomes provide one such mechanism [15].

Exosomes are 40-100 nm extracellular vesicles which participate in a variety of physiological and pathological processes [16]. Their cargo includes proteins, mRNAs and miRNAs, which can be shuttled between cells to facilitate intercellular communication [17]. An increasing body of evidence shows that exosomes play critical roles in several aspects of cancer progression, ranging from proliferation, invasion, metastasis, pre-metastatic niche formation and metastatic organotropism [18-23]. Furthermore, stromal exosomes containing non-coding RNAs have been shown to increase chemo- and radio-resistance by modifying gene expression in recipient cancer cells [15].

With a focus on CAFs as stromal drivers of tumor progression, we aimed to investigate the exosome-mediated crosstalk between CAFs and cancer cells. To achieve this, we derived paired normal and cancer-associated colorectal fibroblasts from human donors, isolated exosomes, and profiled their miRNA content using a high sensitivity direct detection array (Nano-String). Here, for the first time, we identify a novel stromal exosome signature in CRC, as part of a prospective biomarker study. Furthermore, we reiterate the importance of stromal miR-21 in CRC progression using an orthotopic murine model and demonstrate that one of the mechanisms of miR-21 transfer between stromal and cancer compartments is mediated by exosomes.

## RESULTS

### Characterization of fibroblast exosomes

**Figure 1:**
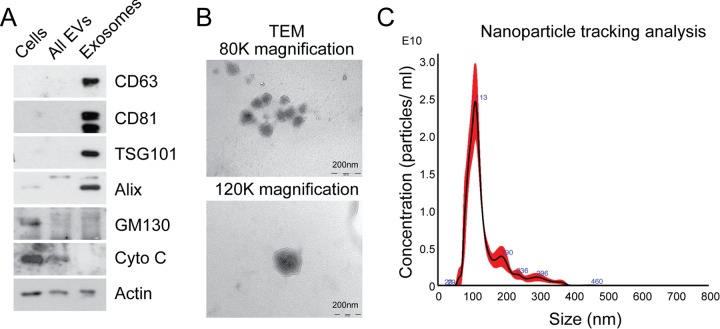
Characterization of exosomes isolated by differential ultracentrifugation (**A**) Western blot analysis to assess expression of exosomal markers in MRC5 exosomes. “Cells” refers to total cellular protein, “all EVs” refers to the total vesicular fraction isolated by a single ultracentrifugation of conditioned medium at 100 000 *g*, and “exosomes” refers to small extracellular vesicles isolated by filtration and serial centrifugation. The exosomal fraction is enriched in tetraspanins (CD63 and CD81), endosomal markers (Alix and TSG101) and does not contain Golgi (GM130) or mitochondrial (cytochrome C) markers. Actin was used as an equal loading control. (**B**) TEM of MRC5 fibroblast exosomes at 80 000x and 120 000x demonstrating homogenous, cup-shaped vesicles with size in the order of 100 nm. Scale bar represents 200 nm in both panels. (**C**) Nanoparticle tracking analysis of MRC5 fibroblast exosomes represented as size vs. concentration.

In order to isolate exosomes from MRC5 fibroblasts, differential ultracentrifugation was performed, producing an exosome pellet which was enriched in vesicle-associated tetraspanins (CD63 and CD81), endosomal proteins (TSG101 and Alix), and devoid of organelle-specific markers such as GM130 (Golgi) and cytochrome C (mitochondria; Fig. 1A). Unfixed MRC5 exosomes visualized by transmission electron microscopy (TEM) demonstrated a uniformly circular morphology with size distribution 40-120 nm (80 000x) and at higher magnification (120 000x) the lipid bilayer structure was clearly seen (Fig. 1B), in keeping with previous descriptions [16]. Nanoparticle tracking analysis (NTA) confirmed a modal size of 113+/−1.3 nm and exosome concentration of 1.57+/−0.16 × 10^12^/ml, which corresponded with a protein concentration of 0.50+/−0.04 μg/μl (Fig. 1C; data not shown). Bioanalyzer analysis of exosomal RNA showed presence of small RNAs and paucity of 18s and 25s ribosomal RNA subunits when compared to cellular RNA (Supplementary Fig. 1), in keeping with previous findings [24].

This meets criteria set by the International Society for Extracellular Vesicles for characterizing extracellular vesicles [25]. Furthermore, our exosome isolation and characterization protocol was assigned an Extracellular Vesicle (EV) Metric of 77% which is in the 99^th^ percentile for all experiments on the same sample type [26]. Finally, the exosome preparations described here could potentially contain other extracellular vesicle populations but given the enrichment of endosomal markers and size distribution, the predominant vesicle type is likely to be exosomes.

### Transfer of fibroblast exosomes to CRC cells occurs and results in increased miRNA levels

**Figure 2:**
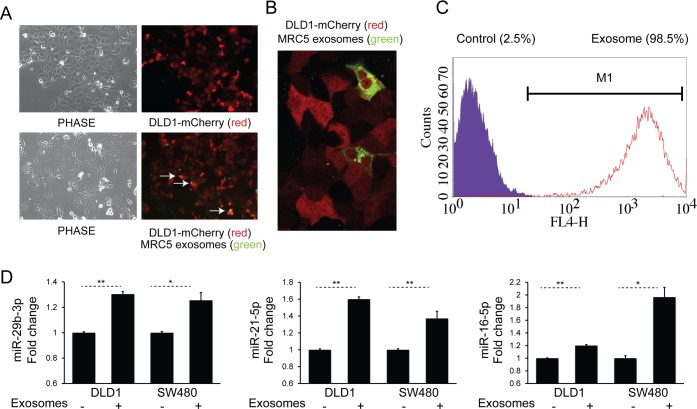
Fibroblast exosomes are taken up by CRC cells resulting in increased miRNA levels (**A**) Culture of mCherry-tagged DLD1 cells (red) in the absence (*top*) or presence (*bottom*) of DiO-labelled MRC5 exosomes (green), visualized by fluorescence microscopy (10x). Co-localization of exosomes with cells is demonstrated by arrows. (**B**) Culture of mCherry-DLD1 cells with DiO-labelled MRC5 exosomes visualized by confocal microscopy (60x), demonstrating the presence of exosomes within cells. (**C**) Flow cytometry of DLD1 cells (control) and DLD1 cells co-cultured with MRC5 exosomes (exosome). The proportion of cells under the M1 region is given as a percentage. (**D**) Co-culture of MRC5 exosomes with DLD1 and SW480 cells with resultant increase in miR-29b-3p, miR-21-5p and miR-16-5p. Data is presented as mean +/− SEM. Paired t-test: ^*^ *p*<0.05, ^**^ *p*<0.01, ^***^ *p*<0.001.

We next sought to examine cellular uptake of fibroblast exosomes by CRC cells. To achieve this, DiO-labeled MRC5 exosomes (green) were co-cultured with DLD-1 cells stably expressing mCherry (red) for 24 h. Fluorescence microscopy showed co-localization of exosomes with cells (Fig. 2A) and confocal imaging confirmed their intracellular location (Fig. 2B). In addition, using flow cytometry, we were able to quantify the extent of exosome uptake (Fig. 2C). Subsequently, to investigate whether fibroblast exosomes can alter miRNA levels in target cells, we co-cultured MRC5 exosomes with two different CRC cells, DLD1 and SW480. This led to a consistent and significant increase in several miRNAs (Fig. 2D).

### Fibroblast exosomes have effects on chemotherapy resistance and proliferation

**Figure 3:**
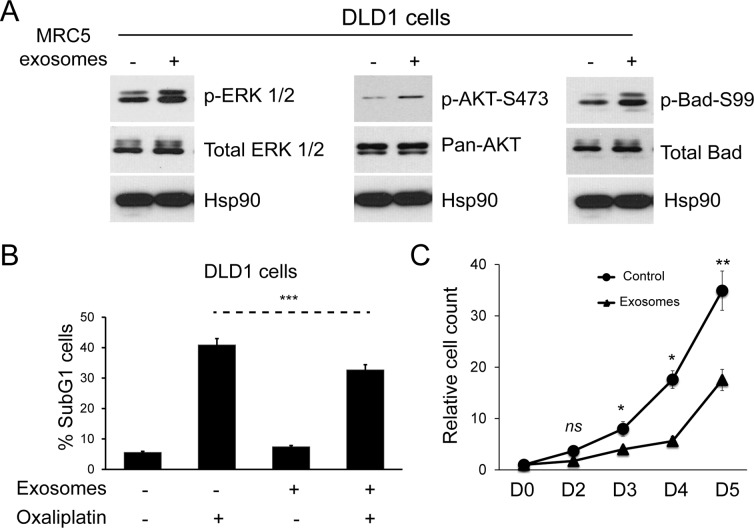
Fibroblast exosomes influence cellular signaling in CRC cells resulting in resistance to chemotherapy and altered proliferation (**A**) Western blot demonstrating ERK (*left*), AKT (*middle*) and Bad activity (*right*) in DLD1 cells in the absence and presence of MRC5 exosomes. MRC5 exosomes induced ERK, AKT and Bad (serine 99) phosphorylation but total ERK, AKT and Bad expression was unchanged. HSP90 was used as an equal loading control. (**B**) Apoptosis of DLD1 cells induced by oxaliplatin in the absence and presence of MRC5 fibroblast exosomes. Fisher's exact test: ^***^ *p*<0.001. (**C**) Proliferation of DLD1 CRC cells in the absence and presence of MRC5 fibroblast exosomes. A significant proliferation defect occurs from day 3 onwards in exosome-exposed CRC cells. Cell counts are relative to day 0, which was given the value 1. Data is presented as mean +/− SEM. Paired t-test: ns – not significant, ^*^ *p*<0.05, ^**^ *p*<0.01, ^***^ *p*<0.001.

Having shown that fibroblast exosomes can be transferred to CRC cells, we investigated effects on cellular signaling pathways and the functional consequences of these. MRC5 exosomes increased ERK phosphorylation in DLD1 cells (Fig. 3A, left).

Similarly, AKT phosphorylation increased, resulting in phosphorylation of a direct AKT target, Bad, at amino acid 99 (Fig. 3A, middle, right) [27]. This was associated with a protective effect on CRC cells in the presence of Oxaliplatin, a first line agent in neoadjuvant and adjuvant treatment of CRC (Fig. 3B, Supplementary Fig. 2) [28]. Contrary to expectation, there was a sustained proliferation defect in DLD1 cells (Fig. 3C).

Having established that exosomes from a normal fibroblast line have functional effects on CRC cells, we sought to characterize the cargo of tissue-specific normal (NOF) and CAF exosomes. To achieve this, we established a library of paired patient-derived primary colorectal NOFs and CAFs from which exosomes could be derived.

### CAFs display a myofibroblastic phenotype and CAF exosomes are also transferred to CRC cells

**Figure 4:**
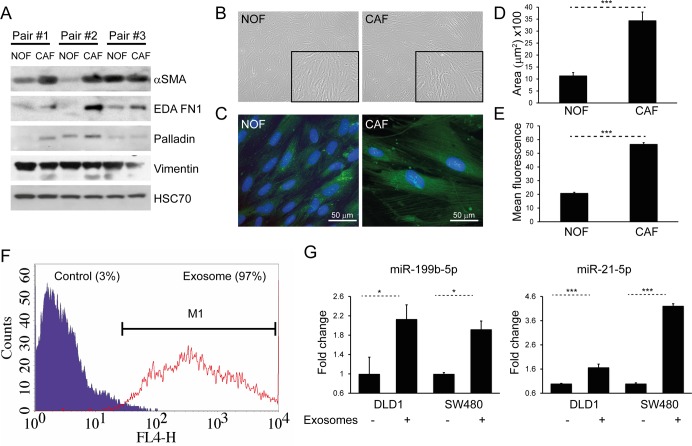
CAFs and NOFs are biochemically and morphologically different and CAF exosomes can also be transferred to CRC cells (**A**) Western blot of paired primary NOFs and CAFs for myofibroblastic markers alpha-smooth muscle actin (α-SMA), fibronectin ED-A (ED-A FN1), palladin and vimentin. HSC-70 was used as an equal loading control. (**B**) Light microscopy of representative primary NOF and CAF cells (10x). (**C**) Fluorescence microscopy demonstrating phalloidin staining of F-actin filaments (green), counterstained with DAPI (blue; 40x). (**D**) Mean surface area and (**E**) intensity of phalloidin staining in a representative NOF-CAF pair. (**F**) Flow cytometry of DLD1 cells (control) and DLD1 cells co-cultured with CAF exosomes (exosome). The proportion of cells under the M1 region is given as a percentage. (**G**) Co-culture of CAF exosomes with DLD1 and SW480 cells with resultant increase in miR-199b and miR-21-5p. Data is presented as mean +/− SEM. Student's t-test (**D, E**) or paired t-test (**F, G**): ^*^ *p*<0.05, ^**^ *p*<0.01, ^***^ *p*<0.001.

In order to demonstrate phenotypic differences between NOFs and CAFs, matched pairs of *ex vivo* colorectal NOFs and CAFs were isolated and characterized using a panel of established experimentally validated markers (Fig. 4A) [29-32]. CAFs occupied a greater surface area than NOFs in two dimensions (Fig. 4B-D), in keeping with previous studies [33]. Intensity of phalloidin staining for F-actin filaments was also significantly higher in CAFs compared to NOFs (Fig. 4C, E), indicating a higher stress fiber density [34]. Similarly to MRC5 exosomes, CAF exosomes were transferred to DLD1 cells (Fig. 4F), resulting in increased miRNA levels (Fig. 4G).

### CAF and NOF exosomes are distinguishable by a specific miRNA signature

**Figure 5:**
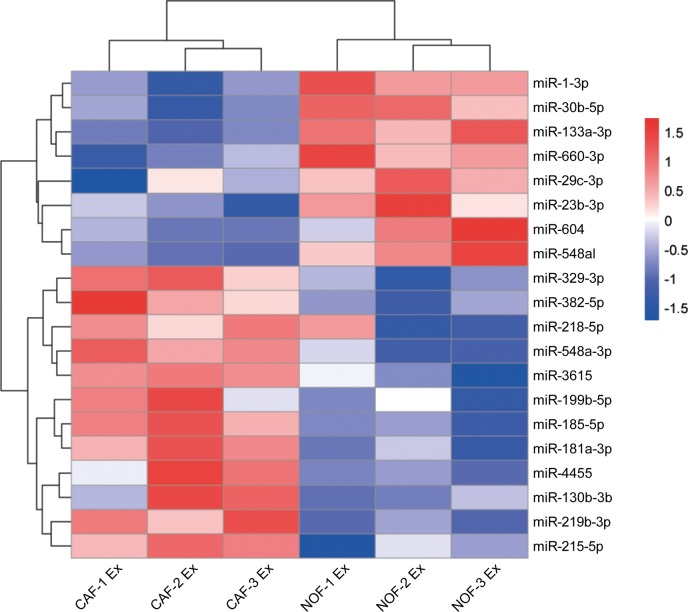
Differential expression of miRNAs in NOF and CAF exosomes Hierarchical cluster analysis of miRNAs in NOF and CAF exosomes. The top 20 most changing miRNAs are shown. Blue-red color scale corresponds with fold changes between −1.5 and +1.5. NOF Ex, normal fibroblast exosome; CAF Ex, cancer-associated fibroblast exosome.

To identify differentially abundant miRNAs, exosomes were isolated from *ex vivo* cultures of primary NOF-CAF pairs and RNA subjected to NanoString assay. Hierarchical cluster analysis of NanoString data separated NOF and CAF exosomes according to miRNA expression, with nine of the 20 most-changing miRNAs less abundant in CAF exosomes and 11 more abundant (Fig. 5, Supplementary Fig. 3). To extend the panel of miRNAs beyond these, we established stringent criteria such that candidate miRNAs had to be: (i) oncogenic, (ii) stromal in origin, (iii) abundant in exosomes and (iv) enriched in exosomes. Ten experimentally validated oncomirs were selected: miR-21, miR-135b, miR-20a/20b, miR-19b, miR-19a, miR-155, miR-181a, miR-130b, miR-95 and miR-499a [35]. Normalized NanoString counts are shown for three NOF-CAF exosome pairs with respect to these oncomirs (Supplementary Fig. 4).

**Figure 6:**
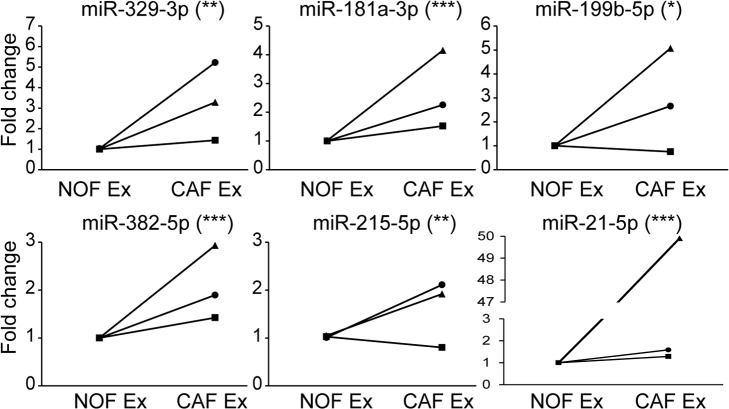
qPCR validation confirms signature of 6 miRNAs more abundant in CAF than NOF exosomes Taqman qPCR results presented as relative fold changes between NOF and CAF exosomal miRNA for each NOF-CAF exosome pair. NOF exosome miRNA level was assigned the value 1 for each NOF-CAF exosome pair (n=3), each of which were analyzed in triplicate. Data is presented as mean +/− SEM. Student's t-test: ^*^ *p*<0.05, ^**^ p<0.01, ^***^ p<0.001.

With a focus on miRNAs which were deliverable in CAF exosomes, we validated six miRNAs (miR-329-3p, miR-181a-3p, miR-199b-5p, miR-382-5p, miR-215-5p and miR-21-5p) which were more rather than less abundant in CAF compared to NOF exosomes (Fig. 6). There was significant correlation between NanoString and RT-qPCR fold changes for NOF-CAF exosomes (*R^2^*=0.81; *p*=0.04), confirming validity of the NanoString platform (Supplementary Fig. 5).

### Exosomal miRNA signature targets multiple cancer-relevant pathways

More than 99% of the total 236 Kyoto Encyclopedia of Genes and Genomes (KEGG) pathways have miRNA sources and targets in man, emphasizing the vast impact of miRNA-mediated regulation within biological pathways. KEGG pathways regulated by miRNAs have tens of thousands miRNA-gene interactions. The number of miRNA-gene interactions related to biological pathways in KEGG for our putatively annotated miRNAs were, respectively, 174: hsa-miR-181a-3p, 299: hsa-miR-199b-5p, 128: hsa-miR-382-5p and 1438: hsa-miR-21-5p. Of these, miR-21 may have the highest regulatory activity of biological pathways by targeting over 1400 genes. We identified 36 KEGG pathways targeted by the combined miRNA signature, including “miRNAs in cancer”, “proteoglycans in cancer”, “colorectal cancer” and “pathways in cancer” (Supplementary Table 1, Supplementary Fig. 6). This was reiterated by Ingenuity Pathway Analysis (Supplementary Tables 2, 3).

A novel approach to identify miRNA-small molecule interactions revealed that this miRNA signature interacts with several drugs utilized in cancer therapy. Of note, we identified a recurrent association between miR-21 and 5-fluorouracil (5-FU), a first line agent in neoadjuvant, adjuvant and palliative CRC (Supplementary Table 4) [28, 36].

### MiR-21 is upregulated in colorectal cancer fibroblasts, enriched in their exosomes and its ectopic overexpression enhances CRC metastasis in an orthotopic CRC murine model

**Figure 7:**
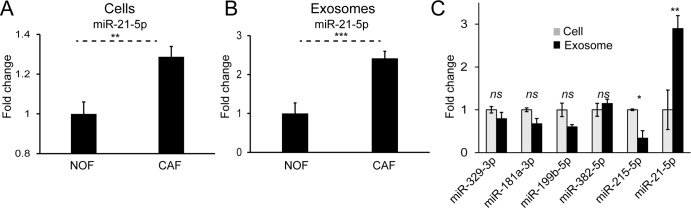
MiR-21 is more abundant in CAF cells and exosomes and enriched in the exosomal compartment (**A**) On a whole-cell level, CAFs express significantly more miR-21 than NOFs. (**B**) CAF exosomes contain significantly more miR-21 than NOF exosomes. Results obtained by Taqman qPCR and presented as mean relative fold changes for each NOF-CAF pair (n=3), analyzed in triplicate. (**C**) NanoString counts normalized by global mean expression for CAF cells and exosomes. Exosomal counts are expressed relative to cellular counts which were assigned the value 1. Data is presented as mean +/− SEM. Student's t-test: ns – not significant, ^*^ p<0.05, ^**^ p<0.01, ^***^ p<0.001.

We have previously shown that miR-21 is a stromal signal in CRC, originating from fibroblasts, and able to influence cancer cells by paracrine mechanisms [9, 10]. Cellular and exosomal profiles of NOFs and CAFs in this study reinforced this, with significantly higher miR-21 levels in CAFs compared to NOFs (Fig. 7A, B). Importantly, we already showed that CAF exosomes contain miR-21 (Fig. 5) and that delivery of CAF exosomes to CRC cells results in increased miR-21 (Fig. 4G). In keeping with this, normalized miRNA counts show an abundance of miR-21 in primary fibroblast exosomes (Supplementary Fig. 4). Further-more, miR-21 was the only miRNA enriched in exosomes compared to parent cells (Fig. 7C). Hence, miR-21 meets all the criteria set above, in that it is oncogenic, stromal in origin, abundant in exosomes and enriched in exosomes, and was therefore the subject of our *in vivo* study.

**Figure 8:**
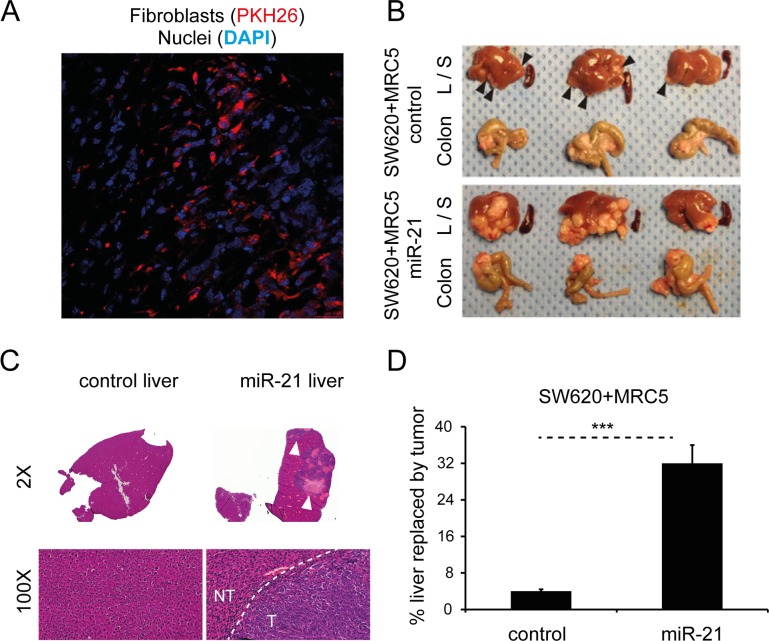
Stromal miR-21 leads to tumor progression in an *in vivo* orthotopic CRC model (**A**) Confocal microscopy of tumor section generated by subcutaneous co-injection of PKH26-labeled MRC5 fibroblasts (red) and CRC cells, counterstained with DAPI (blue; 60x). (**B**) Liver (L), spleen (S) and colon from mice orthotopically injected with SW620 CRC cells and MRC5 control or miR-21-overexpressing fibroblasts. Arrowheads highlight liver metastases. The effect of miR-21-overexpressing cells was to increase the size and number of liver metastases. No splenic metastases were seen in either group. (**C**) Representative liver sections at 2x and 100x magnification. Bulky hepatic metastases are evident in the SW620/MRC5-miR-21 liver (arrowheads; 2x) with a clear histological demarcation between normal liver and metastatic tumor (NT – normal tissue, T – tumor; 100x). (**D**) Percentage liver replacement by metastatic tumor in SW620/MRC5-control (control) and SW620/MRC5-miR-21 (miR-21) mice. Data is presented as mean +/− SEM. Student's t-test: ^***^ *p*<0.001.

Firstly, in order to demonstrate that injected human fibroblasts persist in murine xenografts, we co-injected PKH26-labeled MRC5 cells (red) with CRC cells to form subcutaneous tumors in immunodeficient nude mice. The PKH26 signal was detectable five weeks after injection (Fig. 8A), suggesting that injected fibroblasts persist in the microenvironment of these tumors.

To date, no direct role for stromal miRNAs in promoting metastasis has been shown in an *in vivo* CRC model. In part, this reflects the limitations posed by conventional non-metastatic heterotopic xenografts. Consequently, we next sought to evaluate the *in vivo* activity of miR-21 using an orthotopic CRC model, modified from our previous description [37]. MiR-21 or control non-targeting sequence (control) was stably overexpressed in MRC5 fibroblasts as previously described [9]. Direct cecal co-implantation of SW620 CRC cells with MRC5 fibroblasts stably overexpressing miR-21 (SW620/MRC5-miR-21) resulted in a greater number and size of metastatic tumor deposits in the liver when compared to control (SW620/MRC5-control), equating to eight times more liver replacement by secondary CRC deposits (Fig. 8B-D). No metastases were noted in the spleens of either group. Histological analysis confirmed the presence of colorectal adenocarcinoma in the liver metastases.

## DISCUSSION

In the present study, we isolated and characterized exosomes from fibroblasts, and demonstrated that these are transferable to CRC cells. Importantly, we showed that fibroblast exosomes contain miRNAs, and when transferred, miRNA levels are altered recipient cells, resulting in functional effects on cell cycle and apoptosis. For the first time, we have extracted and characterized paired primary colorectal NOFs and CAFs isolated their exosomes, and profiled the exosomal miRNA cargo using NanoString. This has revealed a novel CAF exosomal signature consisting of miR-329, miR-181a, miR-199b, miR-382, miR-215 and miR-21, which have been shown to regulate multiple cancer-relevant pathways across several tumor types [12, 38-42].

The identification of a stromal exosome signature in CRC has important implications for biomarker development. Firstly, miRNA expression profiles effectively classify cancer into subtypes, and miRNAs have long been proposed as suitable diagnostic and prognostic biomarkers in various cancers [43]. Existing biomarkers for CRC, such as carcinoembryonic antigen, are known to be poorly sensitive, particularly in the diagnostic setting [44]. Secondly, the stroma is a key determinant of cancer development and progression [4, 7]. We and others have demonstrated the value of stromal miRNAs as prognostic markers in CRC [10, 45]. In addition, the stromal compartment of a tumor is genetically more stable than the cancer compartment [46]. Therefore, there should be less variability in stromal miRNA profiles compared to cancer cell or whole tumor profiles, increasing reproducibility across patients. Lastly, exosome encapsulated miRNAs have proven to be representative of the tumor, protected from degradation, and disseminated in the circulation, which improves their utility as circulating biomarkers and liquid biopsy material [47-51].

From our profiling data, we were particularly interested in identifying miRNAs of stromal origin with relevance in CRC. Considering that we were looking for miRNAs that could be transmitted to cancer cells from the stroma, we focused on those with known oncogenic effects [35]. Additionally, the selected miRNAs had to be abundantly expressed in CAF exosomes such that significant amounts could be delivered to CRC cells. We found that miR-21 met all these criteria (Supplementary Fig. 4, Fig. 4G).

MiR-21 is widely accepted to have oncogenic effects across several tumor types [12, 52]. Its most well described interactions are with the tumor suppressors PTEN and PDCD4 [12, 52-57]. In the context of CRC, Asangani and colleagues demonstrated an inverse correlation between miR-21 and PDCD4 in multiple CRC cell lines, with direct binding to its 3′UTR, leading to increased invasive capability [12]. We and others have previously demonstrated that miR-21 is a stromal signal in CRC, using techniques such as in situ hybridization and laser capture microdissection [9, 10, 58]. This seems to be a generalizable finding spanning different solid tumors [59, 60]. Interestingly, Yeung et al. recently demonstrated the role of stromal exosomes in promoting chemoresistance in ovarian cancer [59]. In this study, exosomes derived from miR-21-over-expressing MEFs (miR-21-MEFs) were transferred to ovarian cancer cells, showing that miR-21 is delivered by exosomes. Subcutaneous ovarian cancer xenografts were then established by co-injection of cancer cells and miR-21-MEFs. Intratumoral taxol injection had significantly less effect on tumor burden in xenografts containing miR-21-MEFs compared to control. We took a similar approach in CRC but used orthotopic xenografts, which provide a more reproducible metastatic model of CRC [61, 62]. Here, we showed that stromal miR-21 is responsible for increased CRC metastasis *in vivo* (Fig. 8B-D). In terms of a mechanism, we previously demonstrated that the secretome of miR-21-overexpressing fibroblasts directly increases proliferative and invasive capacity of CRC cells [9]. We present evidence here that one component of the secretome, exosomes, are abundant and enriched in miR-21 (Fig. 7C, Supplementary Fig. 4). Further-more, transfer of CAF exosomes results in increased miR-21 in recipient CRC cells (Fig. 4G). Based on these observations, we propose that in the CRC microenvironment, CAFs deliver miR-21 to cancer cells in exosomes, promoting metastatic cancer progression. Of course, this is one mechanism of stromal-tumor crosstalk and others may exist in parallel, such as CAF-derived soluble factors (e.g. TGBβ, SDF1) and juxtacrine signaling [63, 64].

In this study, for the first time, we derived paired primary NOFs and CAFs from CRC specimens, isolated their exosomes and interrogated their miRNA cargo. We identified a novel miRNA signature specific to CAF exosomes, consisting of miRNAs which have proven relevance in cancer biology. Finally, we selected miR-21 as an oncogenic stromal signal which is abundant in exosomes, and demonstrated its importance in CRC progression using an orthotopic CRCmodel. Taken together, these results add weight to the evidence implicating exosomal miRNAs (exomiRs) in cancer progression, and particularly point a spotlight on the actions of miR-21.

## MATERIALS AND METHODS

### Patient material

All patients were prospectively recruited as part of an ongoing UK National Institute of Health Research Clinical Research Network study (UKCRN ID 6067; NCT03309722), investigating the molecular pathology of CRC and designed to identify novel biomarkers. Other results and further details from this ongoing study have been previously reported [10, 37, 65, 66]. Study oversight activities and monitoring were performed at an independent clinical research organization. All patients provided written informed consent and the study was approved by the regional research ethics committee. Pathological verification of diagnosis and staging was in accordance with the Association of Coloproctology of Great Britain and Ireland guidelines [67]. Information relating to patient demographics, pre-operative risk, imaging, surgery, pathological features, post-operative management and oncological outcomes were extracted. Exclusion criteria included evidence of a hereditary tumor, presence of multiple tumors, tumors with histologically identified extensive necrosis and tumors with synchronous metastases at presentation. Samples from patients with biopsy proven CRC were obtained fresh at the time of surgery. Patient characteristics are summarized in Table 1. Three consecutive patients, none of whom had received neoadjuvant chemotherapy or radiotherapy, were prospectively included. Immediately following excision of the surgical specimen, 1-2 cm diameter biopsies were taken from the tumor site and from normal colonic epithelium remote to the tumor, complying with the traditional view of resection margins in colorectal cancer surgery [68-70].

**Table 1:** Demographic and clinical characteristics of study patients

Patient ID	CRA-460-14 (1)	CRA-463-14 (2)	CRA-602-15 (3)
**Age (years)**	79	68	79
**Sex**	F	M	M
**Ethnicity**	Caucasian	Caucasian	Caucasian
**Tumor Site**	Rectum	Sigmoid	Sigmoid
**pTNM**	T3N0M0	T4aN0M0	T4aN2M0
**AJCC Stage**	II	II	III
**Dukes’ Stage**	B	B	C1
**Differentiation**	Moderate	Moderate	Well-moderate
**EMVI Status**	Negative	Negative	Negative
**MSI Status**	Negative	Negative	Negative
**Neoadjuvant Treatment**	No	No	No

pTNM, pathological TNM stage (5^th^ edition); AJCC, American Joint Committee on Cancer; EMVI, extramural vascular invasion; MSI, microsatellite instability

### Extraction of primary fibroblasts

We previously described a method for extracting primary fibroblasts from colorectal tissue specimens [71]. Briefly, fresh tissue was collected in 10 ml phosphate buffered saline (PBS) supplemented with 2% (double-strength) penicillin–streptomycin (Penicillin (200 U/mL)–streptomycin (200 μg/mL; Sigma-Aldrich, Saint Louis, USA) and 0.1% (0.25 μg/mL) amphotericin B (Fungizone; Thermo Fisher Scientific, Waltham, USA) and washed 3 times with PBS. Tumor and normal biopsies were divided into 2-3 mm fragments in sterile conditions. Each fragment was attached to one well in a 12-well tissue culture plate containing Dulbecco's Modified Eagle's Medium (DMEM; Sigma-Aldrich, Saint Louis, USA) supplemented with 20% fetal calf serum (FCS), 2% penicillin–streptomycin and 0.1% amphotericin B, and incubated at 37°C/ 5% CO_2_. Growth medium was changed every 72 h. Outgrowth of fibroblasts was typically seen at 4 weeks, when cells were expanded in the standard manner.

### Isolation of exosomes

Exosomes were isolated by differential ultra-centrifugation as previously described [71]. Briefly, fibroblasts were grown to 70% confluency in 12 175 cm^2^ flasks (3-4×10^7^ cells), at which point the growth medium was replaced with equivalent medium containing exosome-free FCS. After 72 h, conditioned medium was harvested and centrifuged at 400 *g* for 5 min at 4°C to pellet cellular contaminant, followed by 2000 *g* for 10 min at 4°C to pellet debris. The supernatant was then passed through a 0.22 μm filter and ultracentrifuged at 100 000 *g* for 75 min at 4°C using the TFT 50.38 rotor (Sorvall, Cambridge, UK). The resulting exosome pellets were pooled, washed with PBS and ultra-centrifuged again at 100 000 *g*. The final exosome pellet was solubilized in 200 μl PBS and stored at −80°C. We submitted all relevant data pertaining to exosome isolation and characterization to the EV-TRACK knowledgebase to assess the quality of our methodology (EV-TRACK ID: JZ2312SI) [26].

### RNA extraction

Total cellular RNA was isolated using the miRNeasy mini kit (Qiagen, Hilden, Germany) and total exosomal RNA using the miRNeasy micro kit (Qiagen, Hilden, Germany), as per the manufacturer instructions. Briefly, 700 μl QIAzol lysis reagent was added to the cells or exosomes and the sample disrupted and homogenized by passage through a 20G needle (cells) or vortexing for 1 min (exosomes). The homogenate was then incubated at room temperature for 5 min. 140 μl chloroform was added to the homogenate, mixed thoroughly and incubated for a further 2 min at room temperature. The mixture was centrifuged at 12 000 *g* for 15 min at 4°C, after which the aqueous phase was collected. One and a half volumes of 100% ethanol were added to the aqueous phase and centrifuged in 700 μl aliquots through an RNeasy Mini (cells) or RNeasy MinElute (exosomes) spin column at 10 000 *g* for 15 s at room temperature. The spin column was then washed twice with RPE buffer (cells), or with RWT followed by RPE followed by 80% ethanol (exosomes). RNA was eluted with 30 μl (cells) or 14 μl (exosomes) nuclease free water. RNA concentration and quality were measured by spectrophotometer (Nanodrop, Thermo Fisher, Waltham, USA) and Bioanalyzer (Agilent, Santa Clara, USA; Supplementary Fig. 1).

### NanoString miRNA profiling

The multiplexed NanoString nCounter miRNA expression assay (NanoString Technologies, Seattle, USA) was used to profile 801 human miRNAs. The assay was performed according to the manufacturer's protocol. Briefly, 100 ng of total RNA (33 ng/μl) was used as input material. A specific DNA tag was ligated to the 3′ end of each mature miRNA, providing exclusive identification for each miRNA species in the sample. The tagging was performed in a multiplexed ligation reaction utilizing reverse complementary bridge oligonucleotides to achieve ligation of each miRNA to its designated tag. All hybridization reactions were incubated at 64°C for 18 h. Excess tags were then removed and the resulting material was hybridized with a panel of fluorescently labeled, bar-coded, reporter probes specific to the miRNA of interest. Abundances of miRNAs were quantified on the nCounter Prep Station by counting individual fluorescent barcodes and quantifying target miRNA molecules present in each sample.

### NanoString data analysis

Raw NanoString miRNA data were quantile-normalized using the voom function as implemented in the limma R package (version 3.30.9). MiRNAs were tested for differential abundance using an empirical Bayes moderated t-test in limma, and p-values were corrected for multiple testing by the positive false discovery rate. Results were then graphically displayed in a heat map showing the 20 largest changes in miRNA expression.

Several other miRNAs (miR-21, miR-17-92 cluster, miR-95, miR-135a/b, miR-155 and miR-499) were selected based on their experimentally proven relevance in colorectal cancer and their roles as oncomirs [35]. Raw NanoString counts were normalized to miR-451, miR-16, miR-30a-5p and miR-30e-5p (a combination of best predicted and experimentally utilized stable endogenous exosomal miRNA controls [48, 49, 72]).

To identify miRNAs that were enriched in exosomes, a global mean normalization method was used because there is no validated panel of miRNAs, which are stably expressed in both exosomal and cellular compartments [73]. For each miRNA of interest, exosomal levels were expressed relative to cellular miRNA levels.

MiRNAs of interest were validated in a distinct biological replicate of the corresponding NanoString sample by RT-qPCR to ensure reproducibility. Relevant data were deposited in the ExoCarta database [74].

### TaqMan qPCR quantitation

TaqMan Advanced (Thermo Fisher, Waltham, USA) miRNA assay reactions were performed to quantitate miRNA expression in cellular and exosomal RNA samples according to manufacturer instructions. The assay reference numbers were as follows: miR-16-5p (477860_mir), miR-21-5p (477975_mir), miR-29b-3p (478369_mir), miR-181a-3p (479405_mir), miR-199b-5p (478486_mir), miR-215-5p (478516_mir), miR-329-3p (478029_mir), miR-382-5p (478078_mir), miR-423-5p (478090_mir). Briefly, for each sample, 4 ng of total RNA (2 ng/μl) was converted into cDNA following poly(A) tailing, adaptor ligation and reverse transcription reactions. A further miR-amp reaction was then carried out on the reverse transcription product. PCR reactions were set up in triplicate using 20X miRNA assays and 2X Fast Advanced Master Mix (Thermo Fisher, Waltham, USA), and performed using the ABI 7500 qPCR (Thermo Fisher, Waltham, USA) instrument with the following cycling parameters: 95°C for 20 s and 40 cycles of 95°C for 3 s/ 60°C for 30 s. Expression levels were normalized to miR-423-5p (endogenous reference gene) calculated from the triplicate of CT values using the ΔΔCT method, and expressed relative to one of the samples that was assigned the value 1. Mean relative levels were calculated for each sample.

### MiRNA pathway analysis

Statistical relevance of potential biological pathways that could be affected by the changes observed in miRNA expression was calculated by the miRPath web-based platform [64]. Putative miRNA target genes were determined using the homology search algorithm microT-CDS with the use of TarBase (database of >600 000 experimentally validated interactions between miRNA and genes) [63]. For microT-CDS, a recommended microT prediction threshold of greater than 0.8 was set. The pathway enrichment analysis of multiple miRNA target genes was performed by comparing the input list to miRNA targets contained in all KEGG pathways. All significantly altered miRNAs were used simultaneously for the pathway enrichment analysis. The significance levels between miRNAs and every pathway were calculated by the Fisher-exact meta-analysis method, with the use of unbiased empirical distribution [75]. The resulting *p*-values signify whether a pathway is targeted by at least one miRNA out of the selected group. *P*-values were adjusted using false discovery rate (FDR) and significance level set to 0.05 [76]. Relationships between miRNAs and small molecules were recovered using miRNet [60], which aggregates interaction data from multiple databases including TarBase, miR2Disease, HMDD, PhenomiR, SM2miR and PharmacomiR.

Additionally, miRNAs of interest were submitted to the Ingenuity Pathway Analysis microRNA Target Filter (QIAGEN, https://www.qiagenbioinformatics.com/products/features/microrna-target-filter). In this analysis, mRNA targets and corresponding canonical path-ways were predicted from a combination of TargetScan, TarBase, miRecords and the Ingenuity Knowledge Base.

### Cell lines and transfection

DLD1, SW480, SW620 and HCT116 colorectal adenocarcinoma and MRC5 fetal lung fibroblast cells were procured from ATCC (Manassas, USA), where they had been characterized by STR profiling. Cells were grown in DMEM supplemented with 10% FCS and 2 mM L-Glutamine, maintained at 37 °C in a humidified atmosphere of 5% CO_2_, and passaged for fewer than 6 months after receipt.

MiR-21 and scrambled control (miR-SCC) miRNA were stably expressed in MRC5 fibroblasts by transfecting precursor miRNA expression plasmids containing IRES-driven GFP reporters and subsequently selecting with puromycin (Genecopoeia, Rockville, USA). Transfections were performed using the Xfect transfection reagent (Clontech Laboratories, Mountain View, USA).

Stable mCherry expression in DLD1 cells was achieved by transfecting mCherry (N2) plasmid and selecting single cell clones with neomycin (Sigma-Aldrich, Saint Louis, USA). Positive clones were identified using fluorescence microscopy. Transfection was performed using the Lipofectamine 3000 transfection reagent (Thermo Fisher, Waltham, USA).

### Fluorescent labeling and transfer of exosomes

Exosomes were isolated using the above method, up to and including the first 100 000 *g* ultracentrifugation. Pooled exosomes were then labeled with the lipophilic dye DiO (absorbance 484 nm, emission 501 nm) or DiD (absorbance 644 nm, emission 665 nm; Thermo Fisher, Waltham, USA) at a working concentration of 1:2500 and incubated at 37°C for 20 min. Labeled exosomes were washed with PBS and centrifuged again at 10 000 *g* for 75 min at 4°C.

DiO-labeled exosomes were co-cultured with DLD1-mCherry cells for 24 h at a concentration of 15 μg/ml, in a 6-well format. Cells were washed with PBS to remove ‘free’ exosomes and viewed at 10x using the Olympus CKX41 microscope (Olympus, Waltham, USA) in green and red channels. Acquired images were split into respective color channels and merged using ImageJ software (NIH; http://rsb.info.nih.gov/ij/).

For flow cytometry, DiD-labeled exosomes were co-cultured with DLD1 cells for 24 h at a concentration of 15 μg/ml, in a 6-well format. Cells were washed with PBS, trypsinized, pelleted and re-suspended in 400 μl of DMEM. The presence of exosomes in DLD cells was assessed by capturing DiD signal in the far red (FL4) channel using a flow cytometer (FACS Calibur, BD Biosciences, San Jose, USA).

To investigate exosome-mediated miRNA changes in recipient cells, exosomes were co-cultured with DLD1 and SW480 CRC cells at a concentration of 15 μg/ml for 24 h in a 6-well format. Control cells were treated with an equivalent volume of exosome-depleted conditioned medium (supernatant remaining after exosome isolation). Twenty-four hours later, cells were washed with PBS to remove ‘free’ exosomes, pelleted and RNA extracted. Cellular levels of miR-16-5p, miR-21-5p, miR-29b-3p and miR-199b-5p were determined by RT-qPCR as appropriate.

### Confocal microscopy

DiO-labeled exosomes were co-cultured with DLD1-mCherry cells for 24 h at 15 μg/ml on 22×22 mm glass microscope slides (VWR International, Fontenay-sous-Bois, France). Cells were washed with PBS and fixed with ice-cold 50/50 acetone-methanol for 5 min after which the fixative was replaced with PBS. Cells were imaged using the Leica TCS SP5 confocal microscope at 60x (Leica Microsystems, Wetzlar, Germany).

### Immunostaining of actin filaments in primary fibroblasts

Primary NOF and CAF cells (pair #2) were seeded on 22×22 mm glass microscope slides (VWR International, Fontenay-sous-Bois, France) and grown to 70% confluency. Cells were washed with PBS, then fixed with ice-cold 50/50 acetone-methanol for 5 min. Cells were then incubated for 30 min with 50 μg/mL phalloidin-FITC (Sigma Aldrich, Saint Louis, USA), followed by 5 min with 1 μg/mL DAPI (Sigma Aldrich, Saint Louis, USA), then washed with PBS. Cells were viewed at 40x using the Olympus CKX41 microscope (Olympus, Waltham, USA). Staining intensity and surface area were measured for nine distinct cells in each field of view using ImageJ software (NIH; http://rsb.info.nih.gov/ij/).

### Western blotting

Cells or exosomes were lysed in 2X Laemmli buffer (4% SDS, 20% glycerol, 10% 2-mercaptoethanol, 0.02% bromophenol blue and 120mM Tris HCl). Proteins were separated under reducing conditions in 8, 10, 12 or 15% SDS-PAGE gels, transferred onto nitrocellulose membranes, detected with AKT (pan; C67E7; 1:1000), phospho-AKT (Ser473; D9E; 1:1000), Bad (D24A9; 1:1000), phospho-Bad (Ser136/99; D25H8; 1:1000), cytochrome C (4272; 1:1000; Cell Signaling Technology, Danvers, USA), Alix (3A9; 1:500), TSG101 (4A10; 1:500; Abcam, Cambridge, UK), CD63 (Ts63; 1:500), CD81 (1.3.3.22; 1:500; Thermo Fisher, Waltham, USA), GM130 (35/GM130; 1:500; BD Biosciences, Oxford, UK), α-SMA, (1A4; 1:2000; Sigma-Aldrich, Saint Louis, USA), FN1-EDA (MAB1940; 1:2000; Merck Millipore, Burlington, USA), palladin (1E6; 1:1000; Novus Biologicals, Littleton, USA), Vimentin (Vim 3B4; 1:1000; Dako, Glostrup, Denmark) primary antibodies and horseradish peroxidase-conjugated secondary antibodies (1:4000; Dako, Glostrup, Denmark). Specific signal was visualized using the SuperSignal West Dura Chemi-luminescent detection kit (Thermo Scientific, Waltham, USA). Membranes were probed for *β*-actin (C4; 1:5000; BD Biosciences, San Jose, USA), HSP90 (1:1000; Cell Signaling Technology, Danvers, USA) or HSC-70, (B-6; 1:2000; Santa Cruz Biotechnology, Dallas, USA), as loading controls.

### Chemoresistance assay

There were four experimental conditions: (i) DLD1 cells, (ii) DLD1 cells treated with oxaliplatin, (iii) DLD1 cells co-cultured with MRC5 exosomes and (iv) DLD1 cells co-cultured with MRC5 exosomes and treated with oxaliplatin. Where applicable, DLD1 cells were co-cultured for 24 h with MRC5 exosomes at a concentration of 15 μg/mL, after which they were washed with PBS to remove ‘free’ exosomes. Where exosomes were not used, exosome-depleted conditioned medium of equivalent volume was used as a control. Oxaliplatin (Sigma-Aldrich, Saint Louis, USA) was used at a working concentration of 200 μM for 24 h, and where applicable, was added to the growth media after the 24 h exosome co-culture.

For subG1 DNA analysis the protocol described by Sayan et al. was used [77]. Briefly, cells were detached, pelleted, fixed with 70% ice-cold ethanol and stored at −20°C overnight. Next morning, cells were centrifuged at 500 g for 5 min and fixation solution discarded. Cells were then re-suspended in 100 μl PBS by gentle vortexing. To stain for DNA, cells were incubated with 0.260 U RNase (in PBS) for 30 min followed by 50 μM propidium iodide (in PBS) for 30 min. SubG1 DNA content was analyzed using a flow cytometer (FACS Calibur, BD Biosciences, San Jose, USA) with duplet exclusion.

### Proliferation assay

DLD1 cells were seeded in quadruplicate at a density of 1000 /well in a 96 well plate. The following day (day 0), MRC5 exosomes were added to achieve a concentration of 15 μg/ml. An equivalent volume of exosome-depleted conditioned medium was added to control cells. Cells were fixed sequentially on days 0, 2, 3, 4 and 5 with ice-cold 50/50 acetone-methanol. On day 5, all cells were stained with 1 μg/mL DAPI, washed with PBS, and the center of each well viewed at 4x using a fluorescence microscope with UV filter (CKX41, Olympus, Waltham, USA). Cell nuclei were counted using ImageJ software (NIH; http://rsb.info.nih.gov/ij/) as previously described [78].

### Transmission electron microscopy

Following exosome isolation, the washed pellet was resuspended in 100 μl ultrapure water and stored at 4°C for up to 7 days prior to processing. Briefly, 10 μl exosome sample was dropped on to Parafilm (Bemis NA, Neenah, USA). A carbon coated formvar copper grid (EM Resolutions, Saffron Walden, UK) was placed on the droplet to immerse its coated side, and incubated for 30 s at room temperature. Excess sample was blotted away using absorbent paper. Similarly, the grid was incubated with 10 μl negative stain (5% ammonium molybdate/ 1% trehalose) for 10 s. Excess negative stain was removed by blotting. The grid was visualized at increasing magnification up to 120 000x using the Tecnai 12 microscope (FEI, Lausanne, Switzerland).

### Nanoparticle tracking analysis

The size distribution of exosomes was measured by nanoparticle tracking analysis (NS300; NanoSight, Amesbury, UK), equipped with an EMCCD camera and a 405 nm diode laser. Silica beads (100 nm diameter; Microspheres-Nanospheres, Cold Spring, NY) were used to calibrate the instrument. Exosome samples were diluted 1:5000 in double filtered PBS to optimize particle number in the field of view. For each sample, five videos, each of 90-seconds duration, were captured. Analysis was performed using the instrument software (NTA 2.3.0.15).

### *In vivo* studies

All mice were housed in a specific pathogen-free facility at the University of Southampton and given a commercial basal diet and water *ad libitum*. To demonstrate persistence of injected fibroblasts *in vivo*, three 6-8 week old female CD-1 nude mice (Charles River, Margate, UK) were co-injected with 5×10^5^ HCT116 CRC cells and 5×10^5^ PKH26-labelled MRC5 fibroblasts into dorsal subcutaneous tissue bilaterally. MRC5 cells were labelled with PKH26 (excitation 551 nm, emission 567 nm; Sigma-Aldrich, Saint Louis, USA) as per manufacturer instructions. At five weeks, animals were humanely euthanized and tumors excised. Tumors were fixed in 2% paraformaldehyde for two hours at 4°C, cryoprotected in 30% sucrose overnight at 4°C, embedded in OCT medium (Electron Microscopy Sciences, Hatfield, USA) and snap frozen before cryosectioning. Mounted sections were imaged at 60x using the Leica TCS SP5 confocal microscope (Leica Microsystems, Wetzlar, Germany).

An orthotopic colorectal cancer model was used as previously described [37]. Briefly, six 6-8 week old female CD-1 nude mice (Charles River, Margate, UK) were anaesthetized under isoflurane anesthesia followed by laparotomy and exteriorization of the caecum. For each animal, 1×10^6^ SW620 human CRC cells and 5×10^6^ human MRC5 fibroblasts (stably expressing miR-21 or scrambled sequence control miRNA; n=3 in each group) were combined with Matrigel (Corning, New York, USA)to a total volume of 200 μl. The cell/ matrix combination was orthotopically co-injected into the submucosal layer of the cecum under magnified vision. The cecum was then returned to the peritoneal cavity and the abdominal wall closed in layers with absorbable suture material. Tumors were allowed to grow for 8-10 weeks until the first mice showed signs of weight loss, at which time all mice were humanely euthanized. Colon, liver and spleen were harvested. Excised tissue was paraffin embedded, stained with hematoxylin and eosin and mounted on to slides, before assessment by a specialist histopathologist who was blinded to the outcome of the experiment. Percentage liver replacement (surface area of tumor relative to total surface area) for multiple sections of each control and “miR-21” liver was measured using ImageJ software (NIH; http://rsb.info.nih.gov/ij/). Values from each section were combined to give an overall mean for each group.

### Statistical analysis

Where individual images (microscopy, western blotting, NTA and flow cytometry) are displayed, these are representative of at least 2 separate experiments. Graphics represent the mean ± SEM, unless otherwise stated. The threshold level of significance was set at 0.05 for all statistical tests. RT-qPCR was performed in triplicate and differences in mean relative values were tested by 2-tailed, paired or unpaired (Student's) t-test, as appropriate. Cell counting was performed in quadruplicate and differences in mean relative counts were compared by paired t-test. Events acquired by flow cytometry were analyzed in a 2×2 contingency table by a 2-tailed Fisher's exact test using the “sum of small p-values” method. Percentage liver replacement was compared by 2-tailed Student's t-test.

## SUPPLEMENTARY MATERIAL TABLES AND FIGURES



## Abbreviations

CRC: colorectal cancer
miRNA: microRNA
CAF: cancer-associated fibroblast
NOF: normal fibroblast

## References

[1] Arnold M, Sierra MS, Laversanne M, Soerjomataram I, Jemal A, Bray F (2017). Global patterns and trends in colorectal cancer incidence and mortality. Gut.

[2] Luengo-Fernandez R, Leal J, Gray A, Sullivan R (2013). Economic burden of cancer across the European Union: a population-based cost analysis. Lancet Oncol.

[3] Simmonds PC, Colorectal Cancer Collaborative Group (2000). Palliative chemotherapy for advanced colorectal cancer: systematic review and meta-analysis. BMJ.

[4] Hanahan D, Weinberg RA (2011). Hallmarks of cancer: the next generation. Cell.

[5] Bhome R, Bullock MD, Al Saihati HA, Goh RW, Primrose JN, Sayan AE, Mirnezami AH (2015). A top-down view of the tumor microenvironment: structure, cells and signaling. Front Cell Dev Biol.

[6] Bhome R, Al Saihati HA, Goh RW, Bullock MD, Primrose JN, Thomas GJ, Sayan AE, Mirnezami AH (2016). Translational aspects in targeting the stromal tumour microenvironment: from bench to bedside. New Horiz Transl Med.

[7] Pietras K, Ostman A (2010). Hallmarks of cancer: interactions with the tumor stroma. Exp Cell Res.

[8] Calon A, Lonardo E, Berenguer-Llergo A, Espinet E, Hernando-Momblona X, Iglesias M, Sevillano M, Palomo-Ponce S, Tauriello DV, Byrom D, Cortina C, Morral C, Barceló C (2015). Stromal gene expression defines poor-prognosis subtypes in colorectal cancer. Nat Genet.

[9] Bullock MD, Pickard KM, Nielsen BS, Sayan AE, Jenei V, Mellone M, Mitter R, Primrose JN, Thomas GJ, Packham GK, Mirnezami AH (2013). Pleiotropic actions of miR-21 highlight the critical role of deregulated stromal microRNAs during colorectal cancer progression. Cell Death Dis.

[10] Bullock MD, Pickard K, Mitter R, Sayan AE, Primrose JN, Ivan C, Calin GA, Thomas GJ, Packham GK, Mirnezami AH (2015). Stratifying risk of recurrence in stage II colorectal cancer using deregulated stromal and epithelial microRNAs. Oncotarget.

[11] Volinia S, Calin GA, Liu CG, Ambs S, Cimmino A, Petrocca F, Visone R, Iorio M, Roldo C, Ferracin M, Prueitt RL, Yanaihara N, Lanza G (2006). A microRNA expression signature of human solid tumors defines cancer gene targets. Proc Natl Acad Sci USA.

[12] Asangani IA, Rasheed SA, Nikolova DA, Leupold JH, Colburn NH, Post S, Allgayer H (2008). MicroRNA-21 (miR-21) post-transcriptionally downregulates tumor suppressor Pdcd4 and stimulates invasion, intravasation and metastasis in colorectal cancer. Oncogene.

[13] Toiyama Y, Takahashi M, Hur K, Nagasaka T, Tanaka K, Inoue Y, Kusunoki M, Boland CR, Goel A (2013). Serum miR-21 as a diagnostic and prognostic biomarker in colorectal cancer. J Natl Cancer Inst.

[14] Kanaan Z, Rai SN, Eichenberger MR, Roberts H, Keskey B, Pan J, Galandiuk S (2012). Plasma miR-21: a potential diagnostic marker of colorectal cancer. Ann Surg.

[15] Boelens MC, Wu TJ, Nabet BY, Xu B, Qiu Y, Yoon T, Azzam DJ, Twyman-Saint Victor C, Wiemann BZ, Ishwaran H, Ter Brugge PJ, Jonkers J, Slingerland J, Minn AJ (2014). Exosome transfer from stromal to breast cancer cells regulates therapy resistance pathways. Cell.

[16] Raposo G, Stoorvogel W (2013). Extracellular vesicles: exosomes, microvesicles, and friends. J Cell Biol.

[17] Valadi H, Ekström K, Bossios A, Sjöstrand M, Lee JJ, Lötvall JO (2007). Exosome-mediated transfer of mRNAs and microRNAs is a novel mechanism of genetic exchange between cells. Nat Cell Biol.

[18] Qu JL, Qu XJ, Zhao MF, Teng YE, Zhang Y, Hou KZ, Jiang YH, Yang XH, Liu YP (2009). Gastric cancer exosomes promote tumour cell proliferation through PI3K/Akt and MAPK/ERK activation. Dig Liver Dis.

[19] O'Brien K, Rani S, Corcoran C, Wallace R, Hughes L, Friel AM, McDonnell S, Crown J, Radomski MW, O'Driscoll L (2013). Exosomes from triple-negative breast cancer cells can transfer phenotypic traits representing their cells of origin to secondary cells. Eur J Cancer.

[20] Peinado H, Alečković M, Lavotshkin S, Matei I, Costa-Silva B, Moreno-Bueno G, Hergueta-Redondo M, Williams C, García-Santos G, Ghajar C, Nitadori-Hoshino A, Hoffman C, Badal K (2012). Melanoma exosomes educate bone marrow progenitor cells toward a pro-metastatic phenotype through MET. Nat Med.

[21] Costa-Silva B, Aiello NM, Ocean AJ, Singh S, Zhang H, Thakur BK, Becker A, Hoshino A, Mark MT, Molina H, Xiang J, Zhang T, Theilen TM (2015). Pancreatic cancer exosomes initiate pre-metastatic niche formation in the liver. Nat Cell Biol.

[22] Hood JL, San RS, Wickline SA (2011). Exosomes released by melanoma cells prepare sentinel lymph nodes for tumor metastasis. Cancer Res.

[23] Hoshino A, Costa-Silva B, Shen TL, Rodrigues G, Hashimoto A, Tesic Mark M, Molina H, Kohsaka S, Di Giannatale A, Ceder S, Singh S, Williams C, Soplop N (2015). Tumour exosome integrins determine organotropic metastasis. Nature.

[24] Lässer C, Eldh M, Lötvall J (2012). Isolation and characterization of RNA-containing exosomes. J Vis Exp.

[25] Lötvall J, Hill AF, Hochberg F, Buzás EI, Di Vizio D, Gardiner C, Gho YS, Kurochkin IV, Mathivanan S, Quesenberry P, Sahoo S, Tahara H, Wauben MH (2014). Minimal experimental requirements for definition of extracellular vesicles and their functions: a position statement from the International Society for Extracellular Vesicles. J Extracell Vesicles.

[26] Van Deun J, Mestdagh P, Agostinis P, Akay Ö, Anand S, Anckaert J, Martinez ZA, Baetens T, Beghein E, Bertier L, Berx G, Boere J, Boukouris S, EV-TRACK Consortium (2017). EV-TRACK: transparent reporting and centralizing knowledge in extracellular vesicle research. Nat Methods.

[27] Koh H, Lee KH, Kim D, Kim S, Kim JW, Chung J (2000). Inhibition of Akt and its anti-apoptotic activities by tumor necrosis factor-induced protein kinase C-related kinase 2 (PRK2) cleavage. J Biol Chem.

[28] de Gramont A, Figer A, Seymour M, Homerin M, Hmissi A, Cassidy J, Boni C, Cortes-Funes H, Cervantes A, Freyer G, Papamichael D, Le Bail N, Louvet C (2000). Leucovorin and fluorouracil with or without oxaliplatin as first-line treatment in advanced colorectal cancer. J Clin Oncol.

[29] Garin-Chesa P, Beresford HR, Walker S, Rettig WJ (1990). Immunohistochemical analysis of the A4 and AO10 (gp110) cell-surface antigens of human astrocytoma. Am J Pathol.

[30] Casey TM, Eneman J, Crocker A, White J, Tessitore J, Stanley M, Harlow S, Bunn JY, Weaver D, Muss H, Plaut K (2008). Cancer associated fibroblasts stimulated by transforming growth factor beta1 (TGF-beta 1) increase invasion rate of tumor cells: a population study. Breast Cancer Res Treat.

[31] Salaria SN, Illei P, Sharma R, Walter KM, Klein AP, Eshleman JR, Maitra A, Schulick R, Winter J, Ouellette MM, Goggins M, Hruban R (2007). Palladin is overexpressed in the non-neoplastic stroma of infiltrating ductal adenocarcinomas of the pancreas, but is only rarely overexpressed in neoplastic cells. Cancer Biol Ther.

[32] Liu Y, Hu T, Shen J, Li SF, Lin JW, Zheng XH, Gao QH, Zhou HM (2006). Separation, cultivation and biological characteristics of oral carcinoma-associated fibroblasts. Oral Dis.

[33] De Wever O, Demetter P, Mareel M, Bracke M (2008). Stromal myofibroblasts are drivers of invasive cancer growth. Int J Cancer.

[34] Calvo F, Ranftl R, Hooper S, Farrugia AJ, Moeendarbary E, Bruckbauer A, Batista F, Charras G, Sahai E (2015). Cdc42EP3/BORG2 and Septin Network Enables Mechano-transduction and the Emergence of Cancer-Associated Fibroblasts. Cell Reports.

[35] Schetter AJ, Okayama H, Harris CC (2012). The role of microRNAs in colorectal cancer. Cancer J.

[36] Moertel CG, Fleming TR, Macdonald JS, Haller DG, Laurie JA, Goodman PJ, Ungerleider JS, Emerson WA, Tormey DC, Glick JH, Veeder MH, Mailliard JA (1990). Levamisole and Fluorouracil for Adjuvant Therapy of Resected Colon Carcinoma. N Engl J Med.

[37] Ling H, Pickard K, Ivan C, Isella C, Ikuo M, Mitter R, Spizzo R, Bullock M, Braicu C, Pileczki V, Vincent K, Pichler M, Stiegelbauer V (2016). The clinical and biological significance of MIR-224 expression in colorectal cancer metastasis. Gut.

[38] Sun CC, Li SJ, Zhang F, Pan JY, Wang L, Yang CL, Xi YY, Li J (2016). Hsa-miR-329 exerts tumor suppressor function through down-regulation of MET in non-small cell lung cancer. Oncotarget.

[39] Pekarsky Y, Santanam U, Cimmino A, Palamarchuk A, Efanov A, Maximov V, Volinia S, Alder H, Liu CG, Rassenti L, Calin GA, Hagan JP, Kipps T, Croce CM (2006). Tcl1 expression in chronic lymphocytic leukemia is regulated by miR-29 and miR-181. Cancer Res.

[40] Hou J, Lin L, Zhou W, Wang Z, Ding G, Dong Q, Qin L, Wu X, Zheng Y, Yang Y, Tian W, Zhang Q, Wang C (2011). Identification of miRNomes in human liver and hepatocellular carcinoma reveals miR-199a/b-3p as therapeutic target for hepatocellular carcinoma. Cancer Cell.

[41] Xu M, Jin H, Xu CX, Sun B, Mao Z, Bi WZ, Wang Y (2014). miR-382 inhibits tumor growth and enhance chemosensitivity in osteosarcoma. Oncotarget.

[42] Georges SA, Biery MC, Kim SY, Schelter JM, Guo J, Chang AN, Jackson AL, Carleton MO, Linsley PS, Cleary MA, Chau BN (2008). Coordinated regulation of cell cycle transcripts by p53-Inducible microRNAs, miR-192 and miR-215. Cancer Res.

[43] Iorio MV, Croce CM (2012). MicroRNA dysregulation in cancer: diagnostics, monitoring and therapeutics. A comprehensive review. EMBO Mol Med.

[44] Locker GY, Hamilton S, Harris J, Jessup JM, Kemeny N, Macdonald JS, Somerfield MR, Hayes DF, Bast RC, Asco AS, ASCO (2006). ASCO 2006 update of recommendations for the use of tumor markers in gastrointestinal cancer. J Clin Oncol.

[45] Nishida N, Nagahara M, Sato T, Mimori K, Sudo T, Tanaka F, Shibata K, Ishii H, Sugihara K, Doki Y, Mori M (2012). Microarray analysis of colorectal cancer stromal tissue reveals upregulation of two oncogenic miRNA clusters. Clin Cancer Res.

[46] Allinen M, Beroukhim R, Cai L, Brennan C, Lahti-Domenici J, Huang H, Porter D, Hu M, Chin L, Richardson A, Schnitt S, Sellers WR, Polyak K (2004). Molecular characterization of the tumor microenvironment in breast cancer. Cancer Cell.

[47] Nedaeinia R, Manian M, Jazayeri MH, Ranjbar M, Salehi R, Sharifi M, Mohaghegh F, Goli M, Jahednia SH, Avan A, Ghayour-Mobarhan M (2017). Circulating exosomes and exosomal microRNAs as biomarkers in gastrointestinal cancer. Cancer Gene Ther.

[48] Ogata-Kawata H, Izumiya M, Kurioka D, Honma Y, Yamada Y, Furuta K, Gunji T, Ohta H, Okamoto H, Sonoda H, Watanabe M, Nakagama H, Yokota J (2014). Circulating exosomal microRNAs as biomarkers of colon cancer. PLoS One.

[49] Matsumura T, Sugimachi K, Iinuma H, Takahashi Y, Kurashige J, Sawada G, Ueda M, Uchi R, Ueo H, Takano Y, Shinden Y, Eguchi H, Yamamoto H (2015). Exosomal microRNA in serum is a novel biomarker of recurrence in human colorectal cancer. Br J Cancer.

[50] Mitchell PS, Parkin RK, Kroh EM, Fritz BR, Wyman SK, Pogosova-Agadjanyan EL, Peterson A, Noteboom J, O'Briant KC, Allen A, Lin DW, Urban N, Drescher CW (2008). Circulating microRNAs as stable blood-based markers for cancer detection. Proc Natl Acad Sci USA.

[51] Webb S (2016). The cancer bloodhounds. Nat Biotechnol.

[52] Frankel LB, Christoffersen NR, Jacobsen A, Lindow M, Krogh A, Lund AH (2008). Programmed cell death 4 (PDCD4) is an important functional target of the microRNA miR-21 in breast cancer cells. J Biol Chem.

[53] Meng F, Henson R, Wehbe-Janek H, Ghoshal K, Jacob ST, Patel T (2007). MicroRNA-21 regulates expression of the PTEN tumor suppressor gene in human hepatocellular cancer. Gastroenterology.

[54] Zhang JG, Wang JJ, Zhao F, Liu Q, Jiang K, Yang GH (2010). MicroRNA-21 (miR-21) represses tumor suppressor PTEN and promotes growth and invasion in non-small cell lung cancer (NSCLC). Clin Chim Acta.

[55] Ma X, Kumar M, Choudhury SN, Becker Buscaglia LE, Barker JR, Kanakamedala K, Liu MF, Li Y (2011). Loss of the miR-21 allele elevates the expression of its target genes and reduces tumorigenesis. Proc Natl Acad Sci USA.

[56] Lu Z, Liu M, Stribinskis V, Klinge CM, Ramos KS, Colburn NH, Li Y (2008). MicroRNA-21 promotes cell transformation by targeting the programmed cell death 4 gene. Oncogene.

[57] Gaur AB, Holbeck SL, Colburn NH, Israel MA (2011). Downregulation of Pdcd4 by mir-21 facilitates glioblastoma proliferation in vivo. Neuro-oncol.

[58] Kang WK, Lee JK, Oh ST, Lee SH, Jung CK (2015). Stromal expression of miR-21 in T3-4a colorectal cancer is an independent predictor of early tumor relapse. BMC Gastroenterol.

[59] Au Yeung CL, Co NN, Tsuruga T, Yeung TL, Kwan SY, Leung CS, Li Y, Lu ES, Kwan K, Wong KK, Schmandt R, Lu KH, Mok SC (2016). Exosomal transfer of stroma-derived miR21 confers paclitaxel resistance in ovarian cancer cells through targeting APAF1. Nat Commun.

[60] Fan Y, Siklenka K, Arora SK, Ribeiro P, Kimmins S, Xia J (2016). miRNet - dissecting miRNA-target interactions and functional associations through network-based visual analysis. Nucleic Acids Res.

[61] Tseng W, Leong X, Engleman E (2007). Orthotopic mouse model of colorectal cancer. J Vis Exp.

[62] McIntyre RE, Buczacki SJ, Arends MJ, Adams DJ (2015). Mouse models of colorectal cancer as preclinical models. BioEssays.

[63] Orimo A, Gupta PB, Sgroi DC, Arenzana-Seisdedos F, Delaunay T, Naeem R, Carey VJ, Richardson AL, Weinberg RA (2005). Stromal fibroblasts present in invasive human breast carcinomas promote tumor growth and angiogenesis through elevated SDF-1/CXCL12 secretion. Cell.

[64] Kojima Y, Acar A, Eaton EN, Mellody KT, Scheel C, Ben-Porath I, Onder TT, Wang ZC, Richardson AL, Weinberg RA, Orimo A (2010). Autocrine TGF-beta and stromal cell-derived factor-1 (SDF-1) signaling drives the evolution of tumor-promoting mammary stromal myofibroblasts. Proc Natl Acad Sci USA.

[65] Cellura D, Pickard K, Quaratino S, Parker H, Strefford JC, Thomas GJ, Mitter R, Mirnezami AH, Peake NJ (2015). miR-19-Mediated Inhibition of Transglutaminase-2 Leads to Enhanced Invasion and Metastasis in Colo-rectal Cancer. Mol Cancer Res.

[66] Zhang L, Pickard K, Jenei V, Bullock MD, Bruce A, Mitter R, Kelly G, Paraskeva C, Strefford J, Primrose J, Thomas GJ, Packham G, Mirnezami AH (2013). miR-153 supports colorectal cancer progression via pleiotropic effects that enhance invasion and chemotherapeutic resistance. Cancer Res.

[67] ACPGBI (2007). Guidelines for the management of colorectal cancer.

[68] Scott N, Jackson P, al-Jaberi T, Dixon MF, Quirke P, Finan PJ (1995). Total mesorectal excision and local recurrence: a study of tumour spread in the mesorectum distal to rectal cancer. Br J Surg.

[69] Hida J, Yasutomi M, Maruyama T, Fujimoto K, Uchida T, Okuno K (1997). Lymph node metastases detected in the mesorectum distal to carcinoma of the rectum by the clearing method: justification of total mesorectal excision. J Am Coll Surg.

[70] Reynolds JV, Joyce WP, Dolan J, Sheahan K, Hyland JM (1996). Pathological evidence in support of total mesorectal excision in the management of rectal cancer. Br J Surg.

[71] Bhome R, Goh R, Pickard K, Mellone M, Sayan AE, Mirnezami A (2017). Profiling the MicroRNA Payload of Exosomes Derived from Ex Vivo Primary Colorectal Fibroblasts. Methods Mol Biol.

[72] Occhipinti G, Giulietti M, Principato G, Piva F (2016). The choice of endogenous controls in exosomal microRNA assessments from biofluids. Tumour Biol.

[73] D'haene B, Mestdagh P, Hellemans J, Vandesompele J (2012). miRNA expression profiling: from reference genes to global mean normalization. Methods Mol Biol.

[74] Mathivanan S, Simpson RJ (2009). ExoCarta: A compendium of exosomal proteins and RNA. Proteomics.

[75] Bleazard T, Lamb JA, Griffiths-Jones S (2015). Bias in microRNA functional enrichment analysis. Bioinformatics.

[76] Alhasan AH, Scott AW, Wu JJ, Feng G, Meeks JJ, Thaxton CS, Mirkin CA (2016). Circulating microRNA signature for the diagnosis of very high-risk prostate cancer. Proc Natl Acad Sci USA.

[77] Sayan AE, Griffiths TR, Pal R, Browne GJ, Ruddick A, Yagci T, Edwards R, Mayer NJ, Qazi H, Goyal S, Fernandez S, Straatman K, Jones GD (2009). SIP1 protein protects cells from DNA damage-induced apoptosis and has independent prognostic value in bladder cancer. Proc Natl Acad Sci USA.

[78] Sayan AE, Stanford R, Vickery R, Grigorenko E, Diesch J, Kulbicki K, Edwards R, Pal R, Greaves P, Jariel-Encontre I, Piechaczyk M, Kriajevska M, Mellon JK (2012). Fra-1 controls motility of bladder cancer cells via transcriptional upregulation of the receptor tyrosine kinase AXL. Oncogene.

