# Supplementary material for: Exosomal microRNAs derived from colorectal cancer-associated fibroblasts: role in driving cancer progression
Source: Aging (Albany NY). 2017 Dec 28;9(12):2666–94. doi: 10.18632/aging.101355 (PMC5764398; doi:10.18632/aging.101355)
Supplement: Supplementary file 1 [file aging-09-2666-s001.pdf]

SUPPLEMENTARY MATERIAL

Supplementary Table 1. KEGG pathway analysis combining gene targets of miR-329-3p, miR-181a-3p, miR-199b-5p, miR-382-5p, miR-215-5p and miR-21-5p.

KEGG Pathway	*p*-value	Genes	miRNAs
MicroRNAs in cancer	9.24E-30	54	4
ECM-receptor interaction	4.32E-21	19	4
Prion diseases	1.20E-16	7	2
Proteoglycans in cancer	2.58E-09	52	4
Glioma	4.67E-08	24	4
Colorectal cancer	3.86E-06	23	4
Hepatitis B	9.78E-06	39	4
Non-small cell lung cancer	1.91E-04	20	4
Pathways in cancer	1.91E-04	79	4
Bladder cancer	3.54E-04	18	4
Endometrial cancer	3.54E-04	19	4
Pancreatic cancer	4.51E-04	24	4
Lysine degradation	5.24E-04	14	2
FoxO signaling pathway	1.01E-03	38	4
Fatty acid elongation	1.04E-04	7	2
PI3K-Akt signaling pathway	1.22E-03	71	4
Focal adhesion	1.31E-03	52	4
Central carbon metabolism in cancer	1.39E-03	20	4
Chronic myeloid leukemia	1.50E-03	24	4
Melanoma	1.63E-03	22	4
Thyroid hormone signaling pathway	3.61E-03	29	4

ErbB signaling pathway	3.97E-03	23	4
Prostate cancer	4.21E-03	27	4
Small cell lung cancer	4.21E-03	25	4
Thyroid cancer	6.83E-03	10	4
HIF-1 signaling pathway	7.72E-03	29	3
Amoebiasis	1.23E-02	26	4
mTOR signaling pathway	1.65E-02	19	4
Renal cell carcinoma	1.95E-02	19	4
Hippo signaling pathway	3.11E-02	29	4
Prolactin signaling pathway	3.17E-02	18	4
Choline metabolism in cancer	4.39E-02	25	4
Adherens junction	4.46E-02	18	3
MAPK signaling pathway	4.46E-02	51	4
Cell cycle	4.90E-02	27	4

Supplementary Table 2. Ingenuity Pathway Analysis of "Diseases and Disorders" associated with miR-329-3p, miR-181a-3p, miR-199b-5p, miR-382-5p, miR-215-5p and miR-21-5p.

Name	*p*-value	Molecules
Cancer	4.87E-02 - 3.31E-09	6
Organismal Injury and Abnormalities	4.87E-02 - 3.31E-09	6
Reproductive System Disease	4.82E-02 - 3.31E-09	5
Connective Tissue Disorders	4.06E-02 - 1.23E-04	2
Inflammatory Response	4.06E-02 - 5.08E-04	4

Supplementary Table 3. Ingenuity Pathway Analysis of "Molecular and Cellular Functions" associated with miR-329-3p, miR-181a-3p, miR-199b-5p, miR-382-5p, miR-215-5p and miR-21-5p.

Name	*p*-value	Molecules
Cellular Development	2.03E-02 - 3.67E-05	4
Cellular Growth and Proliferation	2.03E-02 - 3.67E-05	4
Cell Morphology	4.17E-02 - 5.62E-04	2
Cell-To-Cell Signaling and Interaction	7.84E-03 - 5.62E-04	2
Cellular Movement	3.62E-02 - 8.42E-04	2

Supplementary Table 4. Network analytics for miRNA-small molecule interactions for miR-329-3p, miR-181a-3p, miR-199b-5p, miR-382-5p, miR-215-5p and miR-21-5p.

miRNA	Molecule	Pubchem ID	Experiment	Pubmed ID
hsa-mir-21-5p	5-fluorouracil	3385	Microarray	21506117
hsa-mir-21-5p	5-aza-2'-deoxycytidine (5-Aza-CdR)	451668	Microarray	22076154
hsa-mir-21-5p	17beta-estradiol (E2)	5757	Microarray	22403704
hsa-mir-21-5p	5-fluorouracil	3385	qRT-PCR	17702597
hsa-mir-21-5p	17beta-estradiol (E2)	5757	Microarray	19528081
hsa-mir-21-5p	3,3'-diindolylmethane (BR-DIM)	3071	qRT-PCR	20724916
hsa-mir-21-5p	5-aza-2'-deoxycytidine (5-Aza-CdR)	451668	Microarray	22076154
hsa-mir-21-5p	17beta-estradiol (E2)	5757	qRT-PCR	22403704
hsa-mir-21-5p	CDF (analogues of curcumin) + gemcitabine	N/A	qRT-PCR	20388782
hsa-mir-21-5p	Cisplatin	84093	qRT-PCR	22475935
hsa-mir-21-5p	Curcumin	969516	Microarray	18347134
hsa-mir-21-5p	Curcumin	969516	qRT-PCR	22363450
hsa-mir-21-5p	Diazobenzene and its derivatives	N/A	Luciferase reporter assay	18712719
hsa-mir-21-5p	17beta-estradiol (E2)	5757	qRT-PCR	19264808
hsa-mir-21-5p	Curcumin	969516	qRT-PCR	20815812
hsa-mir-21-5p	Bisphenol A	6623	Microarray	20417706
hsa-mir-21-5p	Bisphenol A	6623	Microarray	20417706
hsa-mir-21-5p	Bisphenol A	6623	Microarray	22403704
hsa-mir-21-5p	Caudatin	21633059	qRT-PCR	23708208
hsa-mir-21-5p	CDF (analogues of curcumin)	N/A	qRT-PCR	20388782
hsa-mir-21-5p	Gemcitabine	60750	qRT-PCR	24460329
hsa-mir-21-5p	Gemcitabine	60750	qRT-PCR	21738581
hsa-mir-21-5p	Ginsenoside Rh2	119307	Microarray	21372826
hsa-mir-21-5p	Ginsenoside Rh2	119307	qRT-PCR	21372826
hsa-mir-21-5p	Ginsenoside Rh2	119307	Microarray	23152132
hsa-mir-21-5p	Glossy ganoderma spore oil	N/A	qRT-PCR	21842656
hsa-mir-21-5p	Dihydrotestosterone (DHT)	10635	Microarray	20945501
hsa-mir-21-5p	17beta-estradiol (E2)	5757	qRT-PCR	19528081
hsa-mir-21-5p	Hydroxamic acid HDACi LAQ824	N/A	Microarray	16452179
hsa-mir-21-5p	Hydroxychloroquine	3652	Microarray	24121037
hsa-mir-21-5p	Hydroxychloroquine	3652	qRT-PCR	24121037
hsa-mir-21-5p	Marine fungal metabolite 1386A	N/A	Microarray	22159329
hsa-mir-21-5p	Gemcitabine	60750	Northern blot	16762633
hsa-mir-21-5p	Microcystin-LR (MC-LR)	445434	qRT-PCR	22265967
hsa-mir-21-5p	Nicotine	89594	qRT-PCR	24756761
hsa-mir-21-5p	Nicotine	89594	qRT-PCR	21081469
hsa-mir-21-5p	N-methyl-N-nitro-N'-nitrosogua nidine (MNNG)	9576410	qRT-PCR	24821435
hsa-mir-21-5p	5-fluorouracil	3385	qRT-PCR	21506117
hsa-mir-21-5p	All-trans-retinoic acid (ATRA)	444795	Microarray	21131358
hsa-mir-21-5p	Glucocorticoid	N/A	qRT-PCR	22815788
hsa-mir-21-5p	Glucocorticoid	N/A	TaqMan low-density array	22815788

hsa-mir-21-5p	Arsenite	544	qRT-PCR	24004609
hsa-mir-21-5p	Sulindac sulfide	5352624	qRT-PCR	22286762
hsa-mir-21-5p	Sunitinib	5329102	qRT-PCR	25061297
hsa-mir-21-5p	Temozolomide	5394	qRT-PCR	22753745
hsa-mir-21-5p	Matrine	91466	qRT-PCR	22832383
hsa-mir-21-5p	Trastuzumab	N/A	qRT-PCR	22384020
hsa-mir-21-5p	Trastuzumab	N/A	Microarray	22384020
hsa-mir-21-5p	Trichostatin A (TSA)	444732	Microarray	19112422
hsa-mir-21-5p	Trichostatin A (TSA)	444732	Northern blot	19112422
hsa-mir-21-5p	Trimetazidine (TMZ)	21109	qRT-PCR	22842854
hsa-mir-21-5p	Triptolide	107985	qRT-PCR	22957792
hsa-mir-21-5p	Trypaflavine	N/A	qRT-PCR	20529860
hsa-mir-21-5p	Valproate	3121	qRT-PCR	20427269
hsa-mir-21-5p	Dihydrotestosterone (DHT)	10635	qRT-PCR	20945501
hsa-mir-21-5p	Doxorubicin	31703	Microarray	19237188
hsa-mir-21-5p	Enoxacin	3229	qRT-PCR	21368194
hsa-mir-21-5p	Etoposide	36462	Microarray	19633716
hsa-mir-21-5p	Trastuzumab	N/A	Microarray	22384020
hsa-mir-21-5p	Formaldehyde	712	Microarray	21147603
hsa-mir-21-5p	Progesterone	5994	Microarray	22543862
hsa-mir-21-5p	CDF (analogues of curcumin) + gemcitabine	N/A	qRT-PCR	21408027
hsa-mir-21-5p	All-trans-retinoic acid (ATRA)	444795	qRT-PCR	21131358
hsa-mir-21-5p	Arsenic trioxide	14888	qRT-PCR	22072212
hsa-mir-21-5p	O,p'-dichlorodiphenyltrichlo-roethane (DDT)	13089	Microarray	22403704
hsa-mir-21-5p	Prednisone	5865	qRT-PCR	24121037
hsa-mir-21-5p	Morphine	5288826	Microarray	20564181

hsa-mir-21-5p	CDF (analogues of curcumin)	N/A	qRT-PCR	21408027
hsa-mir-21-5p	Prednisone	5865	Microarray	24121037
hsa-mir-21-5p	Polylysine	162282	qRT-PCR	20529860
hsa-mir-199b-5p	Imatinib mesylate	123596	qRT-PCR	20460641
hsa-mir-199b-5p	Imatinib mesylate	123596	TaqMan low-density array	20460641
hsa-mir-199b-5p	Glucose	5793	qRT-PCR	24394957
hsa-mir-199b-5p	4-hydroxynonenal	5283344	Microarray	19022373
hsa-mir-199b-5p	Enoxacin	3229	qRT-PCR	18641635
hsa-mir-181a-3p	Gemcitabine	60750	Microarray	19237188
hsa-mir-181a-3p	Curcumin	969516	qRT-PCR	22510010
hsa-mir-181a-3p	Doxorubicin	31703	Microarray	19237188
hsa-mir-181a-3p	Gemcitabine	60750	Northern blot	16762633
hsa-mir-181a-3p	4-hydroxynonenal	5283344	Microarray	19022373
hsa-mir-181a-3p	Diethylstilbestrol	448537	Microarray	19549897
hsa-mir-215-5p	Trichostatin A (TSA)	444732	Microarray	21971930
hsa-mir-215-5p	Formaldehyde	712	Microarray	21147603
hsa-mir-215-5p	Arsenic trioxide	14888	qRT-PCR	22072212
hsa-mir-382-5p	Morphine	5288826	qRT-PCR	21224041
hsa-mir-382-5p	Vorinostat (SAHA)	5311	Microarray	19513533
hsa-mir-329-3p	Glucose	5793	Microarray	24394957
hsa-mir-329-3p	Gemcitabine	60750	Northern blot	16762633

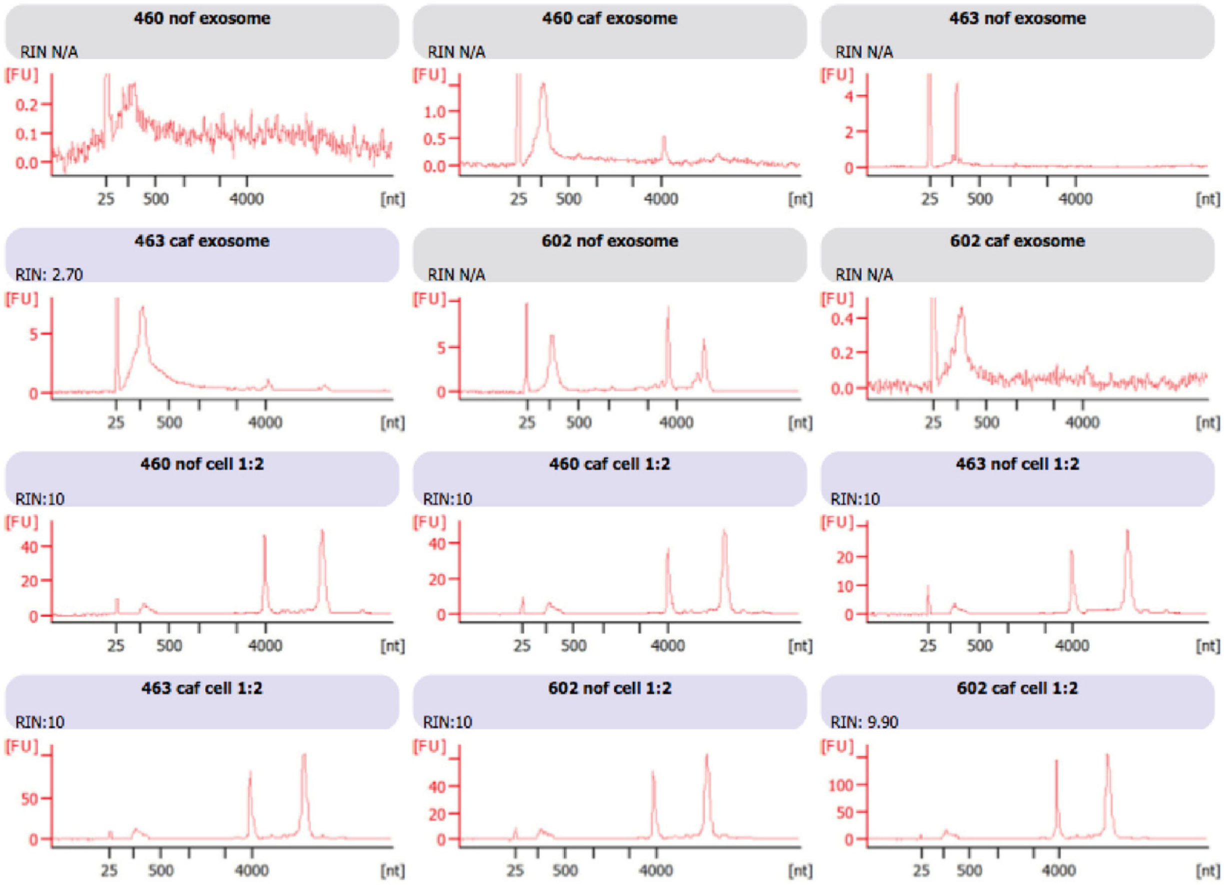

Supplementary Figure 1. Assessment of RNA by Bioanalyzer reveals distinct cellular and exosomal profile. Exosomal RNA lacks the 18s and 28s ribosomal RNA peaks which are seen clearly in total cellular RNA. Therefore, RIN (RNA Integrity Number) is not applicable to exosomal RNA. RIN of all cellular RNA samples was greater or equal to 9.90. Cellular RNA samples were diluted 1:2. The peak at 25 nt represents the marker.

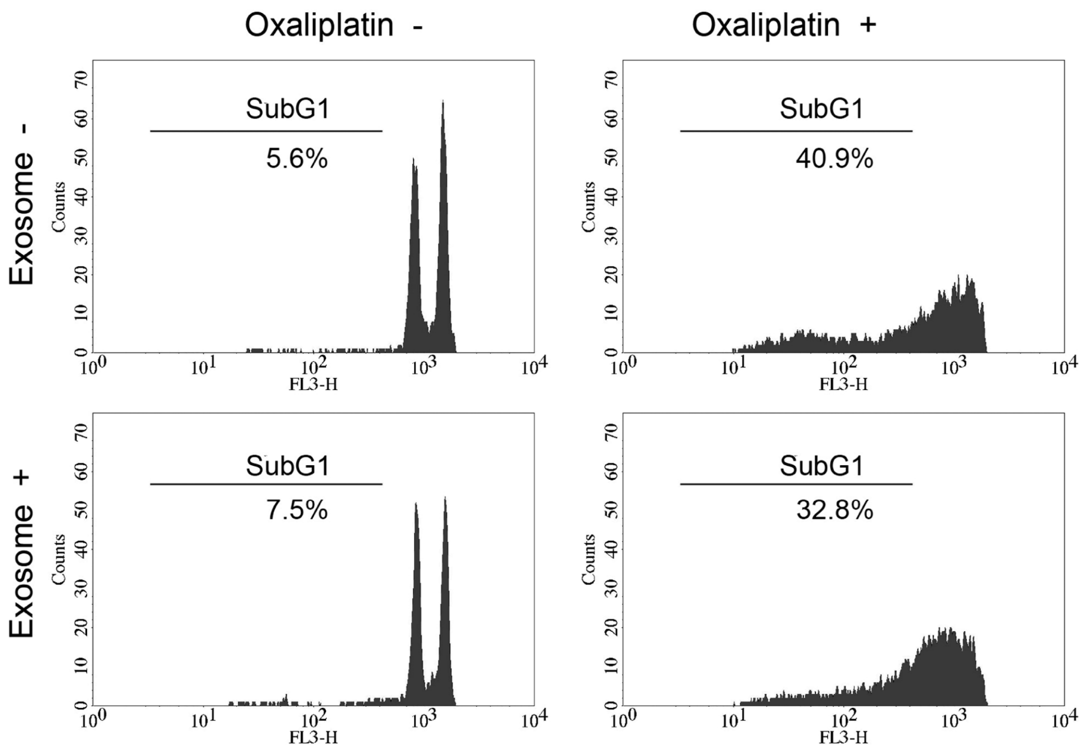

Supplementary Figure 2. SubG1 analysis by flow cytometry demonstrates protective effect of fibroblast exosomes in the presence of oxaliplatin. *Top left*: control DLD1 cells; *top right*: DLD1 cells treated with 200 µM oxaliplatin for 24 h; *bottom left*: DLD1 cells co-cultured with 15 µg/ml MRC5 fibroblast exosomes for 24 h; *bottom right*: DLD1 cells co-cultured with 15 µg/ml MRC5 exosomes for 24 h, then treated with 200µM oxaliplatin for 24 h. Cells registered prior to the G1 peak (subG1) are considered apoptotic.

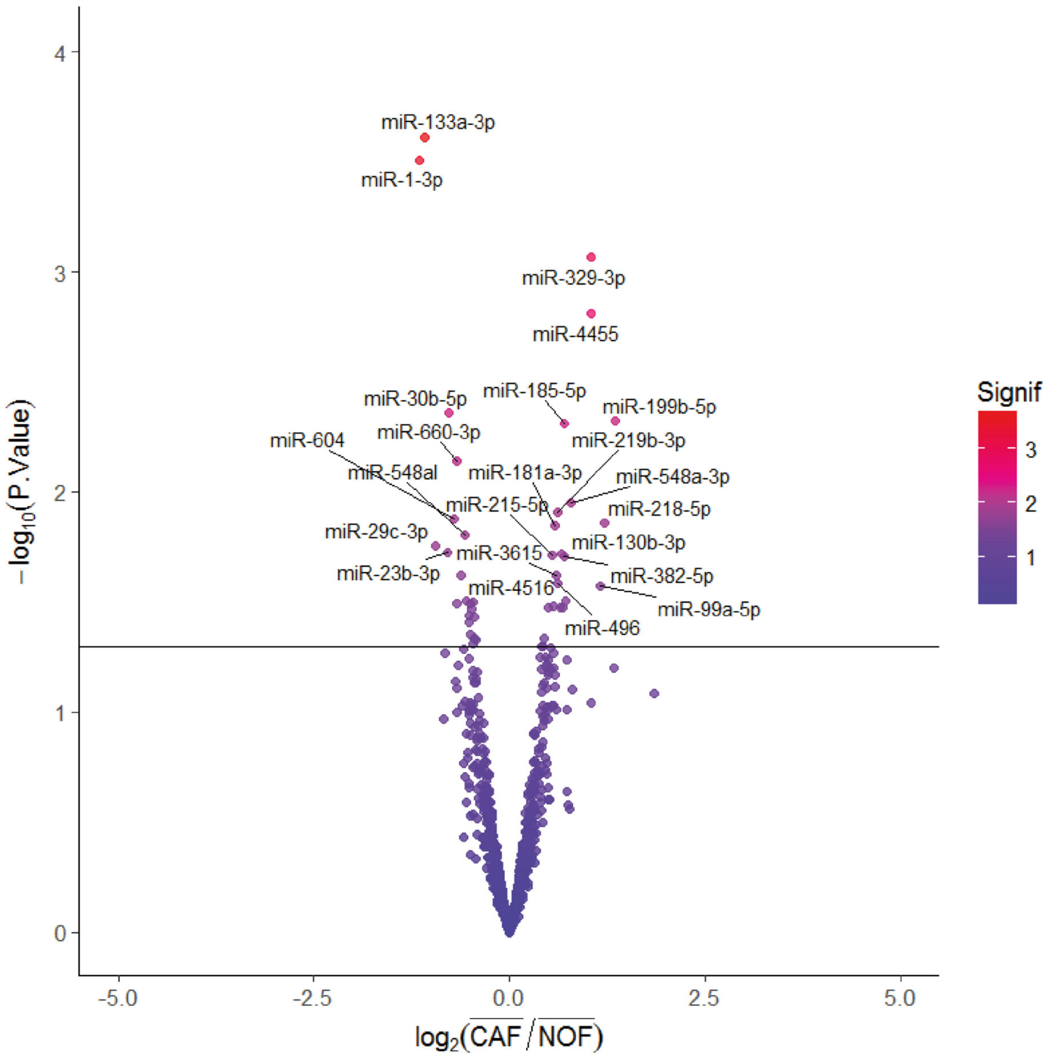

Supplementary Figure 3. CAF and NOF exosomes contain distinct miRNA cargos. Volcano plot displaying miRNAs which are more or less abundant in CAF compared to NOF exosomes (x-axis) against statistical significance (y-axis). This is an alternative representation of data displayed in the previous heat map (Fig. 5). Threshold of statistical significance set at 0.05.

miRNA	460 NOF Ex	460 CAF Ex	463 NOF Ex	463 CAF Ex	602 NOF Ex	602 CAF Ex
miR-21-5p	12132	16327	8216	16316	5404	6330
miR-135b-5p	46	72	31	78	71	1635
miR-20a/b-5p	146	306	89	284	158	210
miR-19b-3p	135	311	115	218	135	232
miR-19a-3p	81	196	85	184	146	141
miR-155-5p	109	110	55	138	92	110
miR-181a-3p	53	107	53	171	71	81
miR-130b-3p	40	61	32	145	67	75
miR-95-3p	43	45	27	62	31	32
miR-499a-5p	26	44	38	53	50	17

Supplementary Figure 4. MiR-21 is abundant and differentially expressed in exosomes from primary colorectal fibroblasts. Heat map of normalized NanoString counts for NOF and CAF exosome samples for ten experimentally validated CRC oncomirs. MiR-21 counts are greater than 5000 for all samples, and consistently higher in CAF exosomes than NOF exosomes.

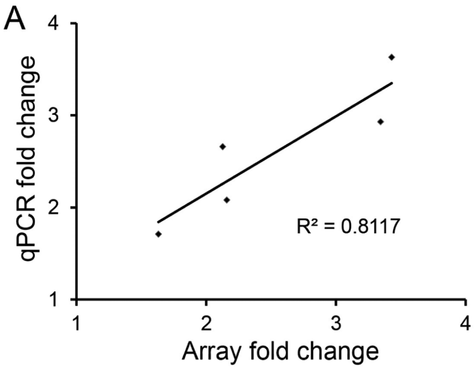

B

miRNA	Array fold change	qPCR fold change
**329-3p**	3.43	3.63
**181a-3p**	2.13	2.66
**199b-5p**	3.35	2.93
**382-5p**	2.16	2.08
**215-5p**	1.63	1.71

Supplementary Figure 5. Nanostring miRNA fold changes correlate with qPCR fold changes. (**A**) Scatter plot of miRNA fold changes between NOF and CAF exosomes determined by NanoString (x-axis) and validated by qPCR (y-axis). Pearson product moment correlation coefficient, R^2 = 0.81; *p*=0.02. (**B**) Numerical values of MiRNA fold changes between NOF and CAF exosomes by Nanostring and qPCR.

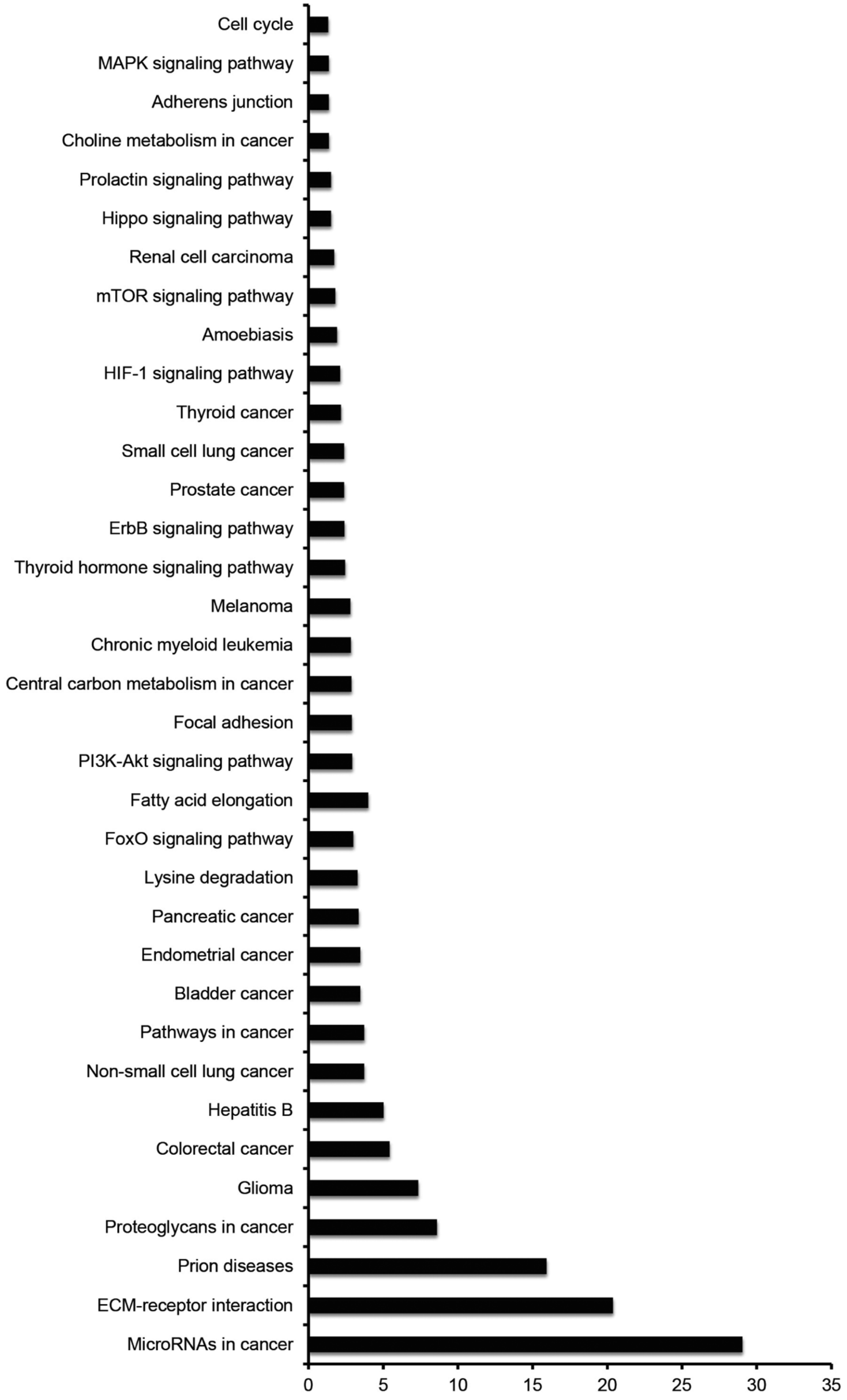

Supplementary Figure 6. CAF-derived exosomal miRNAs converge on multiple cancer-relevant pathways. Statistical significance of 36 KEGG pathways co-regulated by miR-329-3p, miR-181a-3p, miR-199b-5p, miR-382-5p, miR-215-5p and miR-21-5p. Data represented as –log 10 (*p* value). Fisher-exact meta-analysis method with FDR-adjusted *p*-values.
